# A transcriptome resource for the koala (*Phascolarctos cinereus*): insights into koala retrovirus transcription and sequence diversity

**DOI:** 10.1186/1471-2164-15-786

**Published:** 2014-09-11

**Authors:** Matthew Hobbs, Ana Pavasovic, Andrew G King, Peter J Prentis, Mark DB Eldridge, Zhiliang Chen, Donald J Colgan, Adam Polkinghorne, Marc R Wilkins, Cheyne Flanagan, Amber Gillett, Jon Hanger, Rebecca N Johnson, Peter Timms

**Affiliations:** Australian Museum, Australian Museum Research Institute, 6 College Street, Sydney, NSW 2010 Australia; School of Biomedical Sciences, Queensland University of Technology, 2 George Street, Brisbane, Queensland 4001 Australia; Institute of Health and Biomedical Innovation, Queensland University of Technology, 60 Musk Avenue, Kelvin Grove, Queensland 4059 Australia; School of Earth, Environmental and Biological Sciences, Queensland University of Technology, 2 George Street, Brisbane, Queensland 4001 Australia; Systems Biology Initiative, School of Biotechnology and Biomolecular Sciences, University of New South Wales, Sydney, NSW 2052 Australia; Faculty of Science, Health, Education and Engineering, University of the Sunshine Coast, Locked Bag 4, Maroochydore DC, Queensland 4558 Australia; Port Macquarie Koala Hospital, Cnr. Roto Place and Lord St, Port Macquarie, NSW 2444 Australia; Australia Zoo Wildlife Hospital, 1638 Steve Irwin Way, Beerwah, Queensland 4519 Australia; Endeavour Veterinary Ecology Pty Ltd, 1695 Pumicestone Road, Toorbul, Queensland 4510 Australia

**Keywords:** Transcriptome, Koala, *Phascolarctos cinereus*, Koala retrovirus, Alpha amylase, Aldehyde reductase

## Abstract

**Background:**

The koala, *Phascolarctos cinereus*, is a biologically unique and evolutionarily distinct Australian arboreal marsupial. The goal of this study was to sequence the transcriptome from several tissues of two geographically separate koalas, and to create the first comprehensive catalog of annotated transcripts for this species, enabling detailed analysis of the unique attributes of this threatened native marsupial, including infection by the koala retrovirus.

**Results:**

RNA-Seq data was generated from a range of tissues from one male and one female koala and assembled *de novo* into transcripts using Velvet-Oases. Transcript abundance in each tissue was estimated. Transcripts were searched for likely protein-coding regions and a non-redundant set of 117,563 putative protein sequences was produced. In similarity searches there were 84,907 (72%) sequences that aligned to at least one sequence in the NCBI nr protein database. The best alignments were to sequences from other marsupials. After applying a reciprocal best hit requirement of koala sequences to those from tammar wallaby, Tasmanian devil and the gray short-tailed opossum, we estimate that our transcriptome dataset represents approximately 15,000 koala genes. The marsupial alignment information was used to look for potential gene duplications and we report evidence for copy number expansion of the alpha amylase gene, and of an aldehyde reductase gene.

Koala retrovirus (KoRV) transcripts were detected in the transcriptomes. These were analysed in detail and the structure of the spliced envelope gene transcript was determined. There was appreciable sequence diversity within KoRV, with 233 sites in the KoRV genome showing small insertions/deletions or single nucleotide polymorphisms. Both koalas had sequences from the KoRV-A subtype, but the male koala transcriptome has, in addition, sequences more closely related to the KoRV-B subtype. This is the first report of a KoRV-B-like sequence in a wild population.

**Conclusions:**

This transcriptomic dataset is a useful resource for molecular genetic studies of the koala, for evolutionary genetic studies of marsupials, for validation and annotation of the koala genome sequence, and for investigation of koala retrovirus. Annotated transcripts can be browsed and queried at http://koalagenome.org.

**Electronic supplementary material:**

The online version of this article (doi:10.1186/1471-2164-15-786) contains supplementary material, which is available to authorized users.

## Background

The koala (*Phascolarctos cinereus,* Goldfuss 1817) is an arboreal Australian marsupial. The species is of scientific interest because of its unique biological adaptations, evolutionary distinctiveness as well as growing concern about its conservation and the impacts of disease on the health of individuals and populations [1]. Next generation sequencing (NGS) technologies such as whole transcriptome shotgun sequencing (mRNA-Seq) can generate insights into these areas of interest.

### Biological adaptations

The koala feeds almost exclusively on *Eucalyptus* (“gum tree”) foliage [1] which is a relatively poor source of energy and is also high in toxins [2]. Koalas display multiple distinctive anatomical, physiological and behavioural characteristics which are thought to be adaptations to this low energy diet. They are highly selective about the leaves they eat and have powerful jaws and ridged molars to enable efficient mastication [1]. Koalas are hindgut fermenters, and have a caecum that is proportionally the largest of any mammal. This enables them to retain and ferment part of their food for long periods, which aids in digestion as well as the retention and cycling of nitrogen [3]. Koalas produce dry faecal pellets to conserve water, as they seldom drink [4]. The brain is very small relative to body weight, perhaps an adaptation to their low energy nutrition [5]. Additionally, koalas have low metabolic rates, and spend much of the day resting [3]. Finally there are likely to be novel aspects to metabolism of toxins in the koala, with several studies published on koala cytochrome P450 genes [6–9], a family of enzymes with key roles in the oxidative metabolism of a wide range of both xenobiotic and endogenous compounds.

Koalas reach sexual maturity when they are around two years old, with an annual breeding season from October to May. Males use a unique vocal organ [10] to bellow frequently during the breeding season, advertising their presence to each other and to receptive females [1]. As in other marsupials, gestation is brief and is followed by a period of six to eight months growing and developing in the mother’s pouch.

### Evolutionary relationships

Modern marsupials (~270 recent species) are confined to Australasia and the Americas, having diverged from eutherian mammals ~160 million years ago (mya) [11]. The Australasian order Diprotodontia is the most diverse of the seven orders of marsupial mammals. This order includes the endemic Australian family Phascolarctidae, of which *P. cinereus* is the only living representative. The family was more diverse at earlier periods with 18 extinct koala species (in eight genera) known from the fossil record of the last 25 million years [12].

There are currently well-annotated genome sequences available for only three marsupial species (Table 1): the gray short-tailed opossum *(Monodelphis domestica)*[13], the Tasmanian devil *(Sarcophilus harrisii)*[14] and the tammar wallaby *(Macropus eugenii)*[15], the latter also belonging to a family within the Diprotodontia. The koala last shared a common ancestor with the Tasmanian devil ~60 mya and the tammar wallaby ~55 mya [16–18].

**Table 1:** **Characteristics of marsupial species commonly referenced in this study**

Common name	Binomial name	Order	Time since divergence from koala (mya) ^1^	Occurrence	Genome sequence			
					Assembly	N50 ^2^	NCBI assembly ID	No. Ensembl r.72 coding genes
koala	*Phascolarctos cinereus*	Diprotodontia	-	Australia	-	-	-	-
tammar wallaby	*Macropus eugenii*	Diprotodontia	55	Australia	Meug_1.0 ^3^	14.5 kb	GCA_000004035.1	15290
Tasmanian devil	*Sarcophilus harrisii*	Dasyuromorphia	60	Australia	DEVIL7.0	1.8 Mb	GCA_000189315.1	18788
gray short-tailed opossum	*Monodelphis domestica*	Didelphimorphia	75	South America	MonDom5	59 Mb	GCF_000002295.2	21327

^1^According to [16, 17].

^2^For supercontigs/scaffolds.

^3^The Meug_2.0 assembly is available but at the time of writing has not been annotated by either Ensembl or NCBI.

### Disease

There are a number of diseases which affect koala populations, the two most significant being infection with the pathogenic bacteria *Chlamydia* and the koala retrovirus (KoRV). The low genetic variation found in some koala populations [19–21] may pose an additional threat because low genetic diversity in immune genes could reduce the potential response to disease [22]. This means that any new disease could impact on a high proportion of individuals in a population.

#### Chlamydia

The manifestations of chlamydial infection vary and include: conjunctivitis, which can lead to blindness; reproductive tract disease, which can cause infertility in females; respiratory tract disease, which can lead to pneumonia; and urinary tract disease, which can spread to the kidneys and eventually cause death [23].

#### KoRV

KoRV is a gamma retrovirus related to gibbon ape leukemia virus. It is thought to have been introduced relatively recently into koala populations by interspecies transmission [24] although the source remains unknown. Infection with KoRV possibly causes neoplastic disease [24], and may also compromise the immune system, thereby predisposing to other diseases including chlamydiosis [25]. Besides its importance in koala health, KoRV is of interest to virologists because it exists in endogenous (integrated into the germline) and exogenous (infectious) forms [26, 27]. A PCR-based study [28] of KoRV in populations in various parts of Australia found higher prevalence and proviral copy numbers in northern Australia than in southern Australia, and suggested that KoRV endogenisation is ongoing. The 8.4 kb KoRV genome has been sequenced [29] and has the genetic organisation typical of other gamma retroviruses, with 505 bp terminal repeats and three coding regions: *gag*, encoding core and structural proteins; *pol*, encoding reverse transcriptase, protease and integrase; and *env*, encoding coat proteins.

Two subtypes of KoRV (KoRV-A and KoRV-B/KoRV-J) are now recognised, as defined by sequence differences and by the cellular receptor used during infection [30]: KoRV-A uses the sodium-dependent phosphate transporter encoded by the *Pit-1 (SLC20A1)* gene [31], whereas KoRV-B uses the thiamine transporter encoded by the *THTR1 (SLC19A2)* gene [25, 32]. This difference in receptor specificity is reflected in sequence variation in a putative receptor binding region in the envelope protein. KoRV-A has been found in Australian koalas [28, 29, 33], in animals kept in zoos in other countries [34–36], as well as in museum specimens [37]. KoRV-B is the more recently described subtype and has been found in animals at the Los Angeles Zoo [25]. KoRV-J, a subtype of KoRV isolated at a Japanese zoo [32], was described as a novel subtype but is now considered to belong to the KoRV-B clade [30]. It appears that KoRV-B has a more limited distribution than KoRV-A, is perhaps not endogenized, and has invaded the koala population more recently.

### Conservation

At the time of the European settlement of Australia (1788), koalas were widespread in eastern Australia but their current distribution has become highly fragmented. Over most of their distribution, European settlement has had a negative impact due to clearing of habitat, hunting, predation by dogs, injury from cars, and perhaps also introduction of disease [4, 38]. These effects have caused local extinctions, but in some areas (e.g. southern Australia), lack of natural predators and relatively high fecundity has resulted in overpopulation of koalas [1]. Hence, in some areas of Australia koala populations are declining, while in others they are relatively stable, or even increasing, making management strategies for the species complex.

The conservation status of the koala varies across Australia but the national government lists the species as “vulnerable” under the *Environmental Protection and Biodiversity Conservation Act* 1999 for combined populations of the states of New South Wales and Queensland and of the Australian Capital Territory.

### Aims

Our aim was to develop a transcriptome resource that will be useful for future molecular genetic studies of the koala, evolutionary studies of marsupials, as well as for the koala genome sequencing project which is currently in progress. In this study we used *de novo* transcriptome assembly to generate a catalog of koala gene transcripts, quantified and annotated these sequences, compared them to the genomes of other marsupials, and examined the transcription of the koala retrovirus.

## Methods

Our study uses data obtained from two koala individuals. The tissue sampling, RNA preparation and RNA sequencing were done independently for these individuals following the different procedures described separately below. Subsequently the same post-sequencing bioinformatic procedures were applied to both datasets. A summary of all the RNA-Seq libraries produced is given in Table 2.

**Table 2:** **Sequence reads**

Animal	Tissue	Sampling comment	Library	No. raw read pairs	No. trimmed read pairs	No. trimmed unpaired reads	Total trimmed sequence (Gb)
PC	Spleen	Cross section of organ	PC001	58334597	55363111	2335398	10.9
	Liver	Cross section of organ	PC004	113366107	96407712	12468062	18.8
	Uterus	Cross section of organ	PC005	50835874	48175221	2068163	9.5
	Kidney	Mainly renal cortex	PC006	52894485	50152880	2190771	9.9
	Lung	Cross section of organ	PC008	57722900	54667996	2369470	10.8
	Heart	Heart muscle limited penetration	PC009	108773370	103882594	13127007	20.4
	Brain	Neocortex frontal lobe (olfactory lobe not available)	PC010	60678718	57283405	2660545	11.3
	Adrenal gland	Cross section of organ	PC011	54127792	51165338	2338177	10.1
	Total	-	-	556733843	517098257	39557593	101.8
Bi	Bone marrow	-	-	78316465	78163966	152426	14.1
	Kidney	-	-	82942780	82852879	89882	14.9
	Liver	-	-	76971566	76062051	908596	13.8
	Lymph node	-	-	74560669	74384819	175789	13.4
	Salivary gland	-	-	78748770	78670628	78101	14.2
	Spleen	-	-	83176067	83083468	92385	15.0
	Testes	-	-	81482406	81302672	179622	14.6
	Total	-	-	556198723	554520483	1676801	99.9

A study by some of us of the koala immunome, which was based on transcriptomic analysis of a subset of the male individual’s tissues, was recently reported [39].

### Female individual

#### Tissue sampling

A female koala “Pacific Chocolate” (PC; Australian Museum registration number M.45022) from a wild population was euthanized by veterinary staff at the Port Macquarie Koala Hospital following unsuccessful treatment of severe chlamydiosis. Samples from nine tissues (liver, heart, lung, brain, kidney, adrenal gland, spleen, uterus and pancreas) were collected immediately after euthanasia into the gaseous phase of liquid nitrogen (-190°C) to protect the RNA from degradation. Where possible a corer was used to subsample a cross-section of each organ. Multiple small extractions (~10 mg) were performed on tissue samples known to be high in proteins, lipids or nucleases. Samples were stored short term at -80°C until processed for RNA extraction.

#### RNA preparation

Total RNA was extracted using a Qiagen RNeasy Mini kit with Qiashredder columns and “on-column” RNase-free DNase1 digestion to eliminate genomic DNA. Yields of RNA extracted varied between tissue types giving between ~3 μg and ~25 μg total RNA/sample. A260/A280 absorbance ratios were between 2.0 and 2.1. All samples passed RIN evaluation except for the RNA from pancreas which was not processed further.

#### cDNA library preparation and sequencing

For mRNA-Seq sample preparation, the Illumina TruSeq RNA Sample Prep Kit v2 was used according to the manufacturer’s instructions, using 1 μg of total RNA as input. This protocol included steps for selection of polyA-containing RNA, and for enrichment of cDNA fragments with adapters ligated on both ends by using 12 cycles of PCR. The resulting cDNA library insert sizes ranged from 80 bp to 350 bp. The libraries were sequenced as 100 bp paired end (PE) reads using an Illumina HiSeq 2000 and Illumina TruSeq v3 SBS reagents, at the Ramaciotti Centre for Genomics at the University of NSW.

### Male individual

#### Tissue sampling

All animal handling was performed by wildlife veterinarians at Australia Zoo Wildlife Hospital. Samples from seven tissues (bone marrow, kidney, liver, lymph node, salivary gland, spleen, testes) were collected from a single male koala “Birke” (Bi), approximately five years in age, a wild animal which was euthanized as a part of veterinary care following admission to the Hospital because of a dog attack. Samples were immediately stored in 15 mL of RNALater (Ambion) for RNA extraction and stored on ice. All tissue samples were transported to the molecular genetics research facility at Queensland University of Technology and stored at -80°C until further use.

#### RNA preparation

Total RNA was extracted from ~100 mg of starting tissue using the TRIzol chloroform method (Life Technologies) and purified using an RNeasy Midi extraction kit (Qiagen) for each tissue separately. Following RNA extraction, genomic DNA was removed using the Turbo DNA-free kit according to the manufacturers protocol (Life Technologies). The integrity of total RNA was measured as a RIN score measured on an RNA 6000 Nano-chip (Agilent Technologies) and all samples had RIN scores above eight.

#### cDNA library preparation and sequencing

Oligo(dT) magnetic beads were used to isolate messenger RNA from > 20 μg of total RNA for each sample and mRNA was sheared into 200–700 bp fragments. Fragmented RNA was converted into cDNA using SuperScript II reverse transcriptase (Life Technologies) and random hexamer primers (Illumina). Double stranded cDNA was purified with a Qiaquick PCR extraction kit (Qiagen). Fragments underwent end repair and an A-tailing procedure before ligation to Illumina paired-end adapters. These fragments were then size selected (~200 bp) by gel purification. Twelve cycles of PCR were used to increase the concentration of the final cDNA library and sequencing was undertaken using 91 bp paired-end sequencing on a HiSeq 2000 (Illumina), at the Beijing Genomics Institute (BGI)-Shenzhen, Shenzhen, China. Each tissue library was sequenced separately.

#### Sequence trimming and screening

We used the read trimming tool Trimmomatic [40] in paired end mode to clean sequences by applying the following steps. Firstly an ILLUMINILLUMINACLIP step (with parameters seed mismatches = 2, palindrome clip threshold = 40, simple clip threshold = 1) was used to identify and remove matches to Illumina Truseq adapor sequences. Next LEADING and TRAILING steps removed bases from the ends of a read if below a threshold quality of 28. A SLIDINGWINDOW step was used to perform a sliding window trim, with a window size of 4 and a required mean quality of 25. Finally a MINLENGTH step was used to discard sequences shorter than 30b. As well as pairs in which both reads passed filtering, single reads with discarded mates were also retained. We used FastQC [41] to check various measures of sequence quality in both raw and trimmed datasets.

### Transcriptome *de novo*assembly

We constructed single sample and global (all samples from an individual) *de novo* assemblies with two methods, Trinity [42, 43] and Velvet-Oases [44, 45]. For global assemblies only a digital normalization procedure was first applied to the trimmed sequences by using the script normalize_by_kmer_coverage.pl, which is part of the Trinity software package, with parameters “kmer_size = 25 --min_kmer_cov = 2”.

The Velvet-Oases assemblies were run by using the Python wrapper script oases_pipeline.py which accomplishes the following steps. Firstly the hashing program velveth (version 1.2.0.8) was run using as input the trimmed reads (both the read pairs and the “orphaned” reads whose mates were removed by filtering) in fastq format. Secondly, data were assembled into contigs with velvetg (version 1.2.0.8) using k values stepping by 8 from 75 to 91. Thirdly, velveth and velvetg were used to merge individual k results. Finally the program oases (version 0.2.08) was then used to derive transcripts (including putative isoforms, allelic variants and paralogous variants) from the merged contig sequences. The Trinity assemblies were run by using the wrapper script Trinity.pl with default options.

Non-redundant (NR) transcript sets were created by clustering sequences with cd-hit-est version 4.6 [46] with sequence identity threshold set to 1.0 and retaining cluster representatives (the longest sequences).

### Estimation of transcript expression levels and differential expression

For each sample, trimmed sequence reads were aligned to NR transcript sequences using bowtie2 version 2.1.0 [47] with parameters “-X 600 --rdg 6,5 --rfg 6,5 --score-min L,-.6,-.4 --no-discordant --no-mixed -k30 -t”. The resulting alignments were stored in Sequence Alignment/Map (SAM) format and used as input to the program express version 4.0 [48] which estimated transcript expression levels within the sample as fragments per kilobase of exon per million fragments mapped (FPKM).

For each individual we used version 3.4.0 of the BioConductor package edgeR [49] to prepare lists of transcripts whose expression was enriched in a particular library. Our data have no replication and so we used a dispersion value of 0.1 when running the edgeR exactTest function.

### Alignment of transcripts to marsupial genomes

For three marsupial species (tammar wallaby, Tasmanian devil and gray short-tailed opossum; see Table 1), genome and predicted protein sequence data were downloaded for use in locally run sequence alignment programs. Genome sequences were downloaded from NCBI. From the Ensembl (release 72) annotations of these three genomes we downloaded protein sequences (translations of all known or novel Ensembl genes) and cDNA sequences. The program blastx version 2.2.27+ [50] was used to align koala transcript sequences to the Ensembl protein sequences.

The program gmap [51] was used to align koala transcript sequences to the opossum genome and to the Tasmanian devil genome and was run with options “ -d genome --cross-species --batch = 4 --tolerant --prunelevel = 3 --npaths = 0 --nthreads = 10 --format = gff3_gene”. We then used the program intersectBed from the bedtools software package [52] to look for overlap between koala gmap alignment features and Ensembl gene features. As input we used two feature format files: a GFF file with the features produced by gmap with type “gene”, and a second file in GTF format describing the Ensembl gene structure (release 72) for opossum and for Tasmanian devil (downloaded from the Ensembl ftp site). The intersectBed program was run with parameters requiring the same strand for both features, and 100% coverage of the Ensembl feature, and the output filtered in the bash shell to produce a non-redundant list of Ensembl gene identifiers.

### ORF identification, protein sequence extraction and clustering

We used the Trinity plugin script transcripts_to_best_scoring_ORFs.pl with default settings to derive putative protein products from transcript sequences. To remove redundancy, protein sequences were clustered with cd-hit version 4.6 [46] with sequence identity threshold set to 1.0, and cluster representatives (the longest sequences) were saved.

### Protein sequence similarity searches

Protein sequence similarity searches were run on the Barrine HPC cluster at the National Computational Infrastructure Specialised Facility (NCI-SF) in Bioinformatics. The program blastp version 2.2.28+ [50] was run with an expect value cutoff of 1x10^-7^ and output saved in BLAST archive format which was subsequently reformatted as XML and/or tabular text format using the program blast_formatter.

For annotation of koala sequences, cluster representative protein sequences were used as queries in searches of a local copy of the NCBI nr protein sequence database. Alignment information for the best ten subject sequences was retained.

For reciprocal best hit (RBH) analysis we first used the program makeblastdb to construct BLAST databases from koala cluster representative protein sequences, and from sequences of wallaby, Tasmanian devil and opossum proteins predicted by Ensembl. We searched each of the three Ensembl databases with the koala sequences, retaining information about only the single top scoring alignment. Subsequently the three reciprocal searches (i.e. querying the koala sequence database) were performed, again keeping only the top alignment. Results were imported into a custom MySQL database and an SQL query used to retrieve sequence pairs with a RBH relationship.

### Annotation of protein sequences

We used version 2.5.0 of b2g4pipe, the command line interface pipeline script from Blast2GO [53], to map protein BLAST hits to gene ontology (GO) terms and to produce sequence descriptions. A local version of the blast2go relational database was installed for use by b2g4pipe.

### KoRV transcript analysis

To identify KoRV transcripts within the koala Velvet-Oases transcriptome, transcript sequences were searched with the program blastn version 2.2.27+ [50] with parameters “-gapopen 1 –gapextend 1” and using as a query the KoRV genome sequence with database accession [GenBank:AF151794].

To detect KoRV splicing events we used the programs TopHat and Cufflinks. Trimmed read pairs were mapped to the KoRV genome sequence AF151794 with version 2.0.11 of TopHat [54] which was run with the parameter “--b2-sensitive”. For the alignment step TopHat called version 2.1.0 of the Bowtie2 alignment program [47]. KoRV transcript structures were predicted by running version 2.1.1 of the program CuffLinks [55] with default parameters using as input the TopHat alignments.

To reassemble KoRV transcripts we produced a subset of reads which align only with the non-LTR portion of the KoRV genome. Firstly, reads were aligned to two KoRV genome reference sequences (KoRV-A variant with accession [GenBank:AF151794] and KoRV-B variant with accession [GenBank:KC779547]) with version 0.6.2-r126 of the program bwa [56]. Aligned reads were identified by filtering with version 0.1.18 of samtools [57]. Read pairs with either read alignment within an LTR were removed and the resulting set of filtered reads were assembled with Trinity [42, 43] using default settings.

Potential protein products of *gag, pol* and *env* genes were identified by using the program tblastn version 2.2.27+ [50] to search koala transcript sequences from the Velvet-Oases transcriptome as well as from the reassembled KoRV transcriptome, using as queries the KoRV protein sequences with database accessions [GenBank:AAF15097], [GenBank:AAF15098] and [GenBank:AAF15099].

To detect SNPs and small indels we interrogated the bam files produced by the bwa alignment procedure described above. We filtered redundant reads by running the rmdup command of the samtools program [57] using the “-S” flag. We used the samtools mpileup command to format the alignments in “pileup” format. The “-B” flag was used to disable base alignment quality (BAQ) calls. The “bcftools view” command was then used to convert the alignments to Variant Call Format (VCF) [58]. Variant calling programs in common use such as bcftool’s vcfutils.pl assume VCF data is from a single locus with alleles in Hardy-Weinberg equilibrium and so are not appropriate for use in this analysis. We therefore filtered these results requiring a minimum read depth of 100 and that the proportion of reads with the alternate allele be at least 0.1. Filtered SNPs and indels were annotated with version 3.5 of the program SnpEff [59].

### Multiple sequence alignment

Where necessary, input sequences were reformatted with the EMBOSS program seqret [60]. Multiple sequence alignments were created with the program muscle version 3.8.31 [61] and reformatted with version 2.8 of the JalView multiple sequence alignment editor [62].

### Koala genome website

The koala genome website [63] was implemented with version 1.1 of the tripal online genome database construction toolkit [64]. Koala sequence annotations (including GO term assignments and expression levels) were summarized in GFF3 format files. GFF3 files, sequences and BLAST results were loaded into the koala genome database with tools from the tripal toolkit. Online genome browsers are implemented with gbrowse version 2.55 [65] and share the same chado database used by the website.

### Ethical approval

No animal ethics committee (AEC) approval was necessary for this work. The two animals referred to in our work were both euthanased as part of standard veterinary care, as is mentioned in the Methods section. Euthanasia was NOT instigated in order to harvest tissue samples. A relevant Australian state government authority (the Queensland Department of Agriculture, Fisheries and Forestry) makes it clear that AEC approval is not required for "Use of cadavers or samples from animals killed at veterinary clinics or shelters for other (veterinary or management) reasons" (see http://www.daff.qld.gov.au/animal-industries/welfare-and-ethics/using-animals-for-scientific-purposes/what-needs-aec-approval#17141).

## Results

### Sequence assembly

We constructed *de novo* transcriptome assemblies from two individual koalas, “Pacific Chocolate” (PC), a female, and “Birke” (Bi), a male. Eight PC tissues and seven Bi tissues were sampled, and mRNA was prepared and used to generate mRNA-Seq sequence data. The number of sequence read pairs generated from each tissue library ranged from 51-113 million in PC and 75-83 million in Bi, and for all tissues combined was 556 million in each individual. After applying adapter removal and quality control procedures there remained in total, for each individual, over half a billion read pairs plus a much smaller number of unpaired reads, together making up approximately 100 Gb of usable sequence per individual (Table 2).

We performed three sets of *de novo* transcriptome assemblies. In a first set of pilot assemblies (Additional file 1) we investigated two different assembly methods (Velvet-Oases and Trinity). In a second set of assemblies (Additional file 1) we investigated the effect on Velvet-Oases assembly outcomes of reducing the size of the input data with a digital normalization procedure. Finally, based on the pilot assembly results, we chose to construct our global (all tissue libraries) assemblies for PC and for Bi using digital normalization followed by assembly with Velvet-Oases. Normalization reduced the global input size in PC from 1,073,754,107 reads to 86,495,216 reads (8%), and in Bi from 1,110,717,767 reads to 74,146,546 reads (7%). After assembly with Velvet-Oases and then clustering with cd-hit-est we obtained non-redundant sets of 370,030 sequences for PC and 381,958 sequences for Bi (Table 3). The number of Tasmanian devil genes covered was 15,490 for PC and 15,328 for Bi, both of which are appreciably larger than the numbers produced by any of the individual library assemblies. These numbers are also consistent with the number of genes (15,500) which result from merging the lists of all genes covered by the individual non-normalized Velvet-Oases assemblies given in Additional file 1. We conclude that the normalization has not caused significant loss of gene coverage and that the global assemblies represent a large number of genes. The two global Velvet-Oases assemblies were used in all our subsequent analyses.

**Table 3:** **Koala transcriptome assemblies**

Input sequences	No. transcripts	Max. length	Mean length	N50	No. proteins hit ^1^	No. genes ^2^
All PC libraries	370030	82521	1688	3381	17357	15490
All Bi libraries	381958	22721	1124	2531	17078	15328

^1^Total number of distinct protein sequences in best-hit translated BLAST alignments of koala transcripts with Ensembl Tasmanian devil proteins.

^2^Total number of distinct Tasmanian devil genes.

### Alignment of transcripts to marsupial genomes

Koala transcriptome sequences were aligned to the opossum and Tasmanian devil genomes. A large proportion (72%) of PC transcripts could be aligned to the Tasmanian devil genome, although the proportion was slightly lower for Bi (Table 4). As expected, the proportion of reads aligned to the opossum genome is lower (since this species is evolutionarily more distant) with the proportion of aligned reads being 55% and 45% for PC and Bi, respectively. We looked at the extent to which alignment locations overlap the locations of predicted genes. There were a large number of Ensembl genes (nearly 16,000 in each genome) that could be identified this way (Table 5). The vast majority of these were protein-coding genes but other categories of gene were also represented, although this was at a proportionally lower level. The number of Tasmanian devil protein-coding genes with overlapping koala transcripts was consistent with the number of genes previously found using BLAST (Table 3).

**Table 4:** **Alignment of koala transcripts to two marsupial genomes**

Animal	Opossum genome		Tasmanian devil genome	
	No. mapped transcripts	No. unmapped transcripts	No. mapped transcripts	No. unmapped transcripts
PC	204041 (55%)	165989 (45%)	267830 (72%)	102200 (28%)
Bi	172910 (45%)	209048 (55%)	195308 (51%))	186650 (49%)

**Table 5:** **Ensembl genes which overlap with aligned koala transcripts**

Gene category	Total	Overlapping PC transcript(s)	Overlapping Bi transcript(s)	Overlapping either PC or Bi transcript(s)
Opossum				
protein_coding	21327	14624	14170	15584
pseudogene	722	214	211	288
snRNA	843	118	98	138
snoRNA	319	206	184	227
miRNA	412	107	82	127
rRNA	176	22	19	25
misc_RNA	77	10	8	10
Mt_tRNA	21	3	0	3
Mt_rRNA	2	1	0	1
total	23899	15305	14772	16403
Tasmanian devil				
protein_coding	18788	14971	14480	15773
pseudogene	178	114	115	128
snRNA	503	94	57	102
snoRNA	277	202	185	213
miRNA	486	166	113	182
rRNA	87	14	10	14
misc_RNA	113	20	20	21
Mt_tRNA	22	0	0	0
Mt_rRNA	2	0	0	0
total	20456	15581	14980	16433

### Annotation of protein sequences

For each animal, Velvet-Oases transcripts were scanned for likely open reading frames which were translated and then clustered to produce a non-redundant set of protein sequences (Table 6). The two sets of protein sequences were also combined to produce an overall set of 117,563 sequences. Clustered sequences were used as queries in protein BLAST searches of the NCBI nr protein database. These results were then used with blast2go to annotate sequences with gene ontology (GO) terms. In the PC protein sequence dataset there were 23,658 sequences annotated with at least one GO term, while in the Bi set there were 34,524 GO-term annotated sequences.

**Table 6:** **Similarity searching and blast2go annotation of koala NR proteins**

Animal	No. proteins	With nr database protein BLAST hit	With GO term(s) assigned
PC	78208	56872	23658
Bi	63554	47161	34524

The majority (77%) of the top-scoring alignments were to sequences from other marsupials and this was examined in greater detail in a second set of protein BLAST similarity searches. Koala protein sequences were compared with the predicted proteomes of three marsupials, wallaby, devil and opossum (Table 7). In each of the three proteomes there are more than 12,000 sequences which were “hit”, even after a reciprocal best hit (RBH) requirement is applied. We also show the number of genes since multiple proteins can be encoded by a single gene, although this is not common in our dataset. Some of the variation in the number of genes found (smallest number in the wallaby and largest in the opossum) is probably due to differences in the sequence assembly and annotation: the number of Ensembl genes is 15,290 in wallaby, 18,788 devil and 21,327 in opossum. The RBH results are summarized in Additional file 2 and indicate that our transcriptome data represent approximately 15,000 koala genes.

**Table 7:** **Number of sequences (and genes, in parentheses) with which koala protein sequences were aligned in protein BLAST searches of three marsupial proteomes**

Animal	***Macropus eugenii***		***Sarcophilus harrisii***		***Monodelphis domestica***	
	BH	RBH	BH	RBH	BH	RBH
**PC**	12842 (12820)	12206 (12198)	15882 (14548)	14065 (13761)	15391 (15045)	14203 (14101)
**Bi**	12554 (12538)	11770 (11763)	15379 (14259)	13494 (13,269)	14981 (1473)	13589 (13507)
**PC + Bi**	13344 (13318)	12673 (12663)	16957 (15285)	14931 (14457)	16233 (15799)	14922 (14774)

BH: best hit. RBH: reciprocal BH.

### Tissue expression levels

The 100 most abundant transcripts with BLAST annotations in each tissue are listed in a spreadsheet (Additional file 3). We also produced lists of transcripts expressed in a tissue-specific manner (Additional file 4), although this categorization is not robust because our study was not designed to identify differential expression of transcripts. Prominent in the lists of highly expressed sequences are transcripts encoding viral proteins which are derived from KoRV (see below).

### Gene duplications

We used the comparisons of our koala protein sequences with opossum, devil and wallaby protein sequences to identify possible gene duplications by looking for cases where two or more koala sequences share the same subject sequence as their “best hit” in a protein BLAST search. There are several factors that complicate this approach, including sequencing errors, assembly errors, coding region prediction errors, allelic variation, isoforms, and incomplete or inaccurate annotation of reference genomes. Nevertheless there were two putative examples of gene expansion which we investigated.

The most obvious gene duplication candidate was alpha amylase, a gene known to undergo copy number variation in a number of species [66]. In the human genome there are several variants of this gene as well as copy number variation which is subject to positive selection [67]. In both opossum and devil genomes, there is only a single gene annotated by Ensembl, while in the wallaby there are two genes, although both appear incomplete, perhaps because of the low-coverage genome assembly employed. In contrast, in the PC and Bi koala transcriptomes, we found nine and ten distinct full length alpha amylase protein sequences respectively (Additional file 5). Even allowing for the possibility that some of this variation is allelic, this result suggests that there may have been expansion of the alpha amylase gene in koala of up to 5-10 gene copies. The protein sequences of alpha amylase showed between 86% and 99% pairwise sequence identity. Interestingly the koala transcripts are not expressed in the salivary gland sample, and are instead expressed most highly in lung (PC) and in liver, testis and spleen (Bi).

The second gene for which we found convincing evidence for gene family expansion in the koala is an aldehyde reductase gene, encoding aflatoxin aldehyde reductase, which is involved in detoxification of aldehydes and ketones. Ensembl annotations predict two genes in the opossum genome, two in the wallaby genome and one in the Tasmanian devil genome, although we note that the NCBI gene database includes two Tasmanian devil genes. As with the alpha amylase genes, the wallaby sequences appear to be incomplete. In the PC and Bi koala transcriptomes we found seven and eight distinct full length aldehyde reductase protein sequences respectively (Additional file 6). Even allowing for the possibility that some of this variation is allelic, this result suggests that there may have been expansion of the gene of up to 4-8 gene copies. The protein sequences show between 83% and 99% pairwise sequence identity. We speculate that the enzyme has a role in the metabolism of toxins and that the expansion of this gene is part of the koala’s evolutionary adaptation to its diet of *Eucalyptus* leaves.

For both the alpha amylase gene and the aldehyde reductase gene we eliminated the possibility that variations were due to sequencing error by inspecting alignments of sequence reads to the predicted transcripts (analyses not shown).

### Koala retrovirus transcription

KoRV transcripts are present in the transcriptomes of both individuals studied. Due to its importance to koala health and also because of its intrinsic interest, we examined KoRV transcription in more detail.

#### KoRV transcripts in Velvet-Oases assemblies

**Figure 1:**
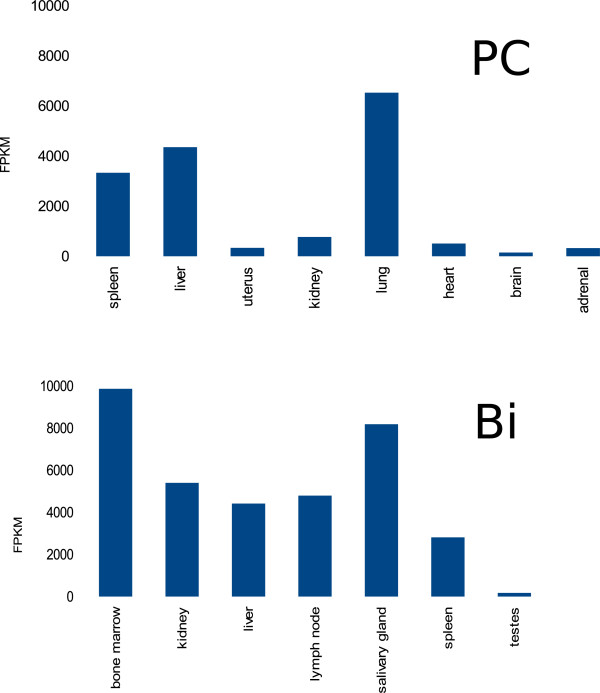
**KoRV expression profile in PC and Bi.**

In both individuals, KoRV transcripts are expressed in all tissues sampled. The expression profiles are shown in Figure 1. In PC, expression is highest in lung, liver and spleen, and in Bi, expression is highest in bone marrow and salivary gland and relatively low in testis. The proportion of the total level of expression (as estimated by summing FPKM values for all transcripts within a tissue) which is due to KoRV, ranges from 0.2% to 0.7% (lung) in PC and 0.01% (testes) to 1.3% (salivary gland) in Bi.

#### Splicing of KoRV transcripts

**Figure 2:**
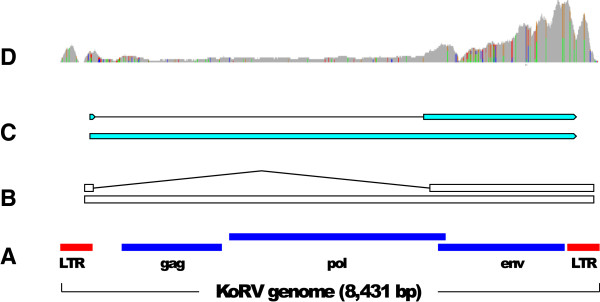
**Transcription of KoRV. A**. Organisation of the KoRV genome [GenBank:AF151794]. **B**. structure of transcripts predicted by Cufflinks. **C**. two transcripts from reassembled KoRV transcriptome (PC spleen). Note LTR sequences excluded from this assembly. **D**. Depth of coverage with reads aligned with bwa (PC spleen).

**Figure 3:**
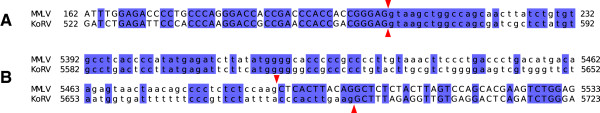
**KoRV splice sites.** Alignment of *env* transcript splice donor **(A)** and acceptor **(B)** regions from Moloney murine retrovirus (MMLV; database accession [GenBank:AF462057]) and koala retrovirus (KoRV; database accession [GenBank:AF151794]). Positions with identical nucleotides are shaded blue. Intronic sequences are in lower case. Red triangles indicate splice sites supported by experimental evidence.

We aligned reads to a reference KoRV genome sequence with Bowtie2 and then used Cufflinks to predict transcript structure and to estimate the abundance of predicted transcripts. Cufflinks predicted two transcripts: a near-full-length unspliced transcript, and an *env* transcript with a single intron (Figure 2). This is consistent with the canonical transcriptional arrangement observed in this class of retrovirus [68]. The *env* transcript intron extends from positions 566-5691 with respect to the KoRV genome sequence we used as a reference (database accession [GenBank:AF151794]). Figure 3 shows a comparison of the *env* splice sites of KoRV and Moloney murine leukemia virus (MMLV), a well-characterised gamma retrovirus. The KoRV donor site is equivalent to that reported for MMLV [69, 70] although the acceptor site is in a slightly different position and corresponds to one of the potential minor acceptor sites discussed in reference [70].

Figure 2D shows the depth of coverage of the KoRV genome by aligned sequence reads. It is apparent that the level of coverage over the *env* gene is higher than over the other genes. There is uneven coverage across *env* which might be explained by sequence variation in the early part of the gene (see below), lowering the efficiency of alignment of reads to that region. Nevertheless, the overall pattern is consistent with a model in which two transcripts are expressed, with the spliced *env* transcript at a higher level than the unspliced transcript. The Cufflinks estimation of FPKM expression level of the *env* transcript is approximately three times higher than that for the longer unspliced transcript.

#### Reassembly of KoRV transcripts

There are 141 PC transcripts and 94 Bi Velvet-Oases transcripts which contain KoRV sequences. However these transcripts do not appear to be correctly assembled, most likely because of the presence of the 505 bp LTR repeat, and so we reassembled KoRV transcripts using only non-LTR reads. The resulting transcripts show much less rearrangement than initial Oases assemblies and are likely to be a better representation of the KoRV transcriptome. In both individuals we were able to reconstruct transcripts over 7 kb in length, corresponding to the non-LTR portion of the KoRV genome. The reassembled KoRV transcriptome includes spliced *env* transcripts consistent with the Cufflinks results. Figure 2C shows selected transcripts from reassembly of transcripts from the PC spleen library.

#### SNP and small indel variations

We compared our KoRV sequences to a published KoRV-A genome sequence ([GenBank: AF151794]) and found 233 sites at which there are single nucleotide polymorphisms (SNPs) or small insertions/deletions (indels). These are summarized in Additional file 7. There are 138 sites in PC only, 61 sites in Bi only, and 34 sites in common. There are 32 sites which also occur in a list of 138 polymorphisms reported in a recent study of KoRV sequence variation in modern and museum koala DNA samples [71]. Most of the variations we observed occur within an individual and are not just a difference between our samples and the reference. There are variations which occur within the *gag, pol* and *env* coding regions and these have various effects, including synonymous and nonsynonymous codon changes, premature termination and frame shifts caused by indels. Of note is a G/A polymorphism at position 6413 which changes Ala to Thr and restores the “CETTG” motif which is implicated in viral fusion activity [72]. To an extent, variation can be attributed to the accumulation of random mutations at multiple endogenised KoRV proviral sites within the genome. However, the number of changes in PC is higher than in Bi (particularly in the *env* gene) suggesting that in PC there is an additional cause of variation.

#### Variation in predicted protein sequences

We examined the reassembled KoRV sequences to find *gag, pol* and *env* sequences, and compared these with published sequences. We found examples of full length *gag*, *pol* and *env* protein sequences in both individuals. In addition there are transcripts with partial sequences and also some with mutations such as frameshifts or short deletions (not shown).

An alignment of full-length (521 amino acid) *gag* protein sequences is shown in Additional file 8. There are three distinct sequences: two sequences are found in both PC and Bi, and one additional sequence is found only in PC. All the sequences are nearly identical with only two amino acid changes between the two most divergent sequences.

We found six distinct full-length (1,127 amino acid) *pol* protein sequences (Additional file 9). One of these is found in both PC and Bi, one in Bi only and four in PC only. As with the *gag* proteins all these sequences are very closely related (over 99% identity) and there are only six amino acid changes between the two most divergent sequences.

In contrast to *gag* and *pol*, the *env* protein sequences show considerably more variation. Most variation occurs between positions 90-150 within a hypervariable region which has been called “variable region A” (VRA) [31]. We found eight distinct full-length (659 to 667 amino acid) sequences which we divided into three groups on the basis of similarity to published sequences. The first group (aligned in Additional file 10) consists of five sequences most closely related to the published KoRV-A *env* sequence with database accession [GenBank:BAM67147] [36]. PC and Bi each share a sequence identical to the published sequence and PC has four additional closely related sequences.

**Figure 4:**
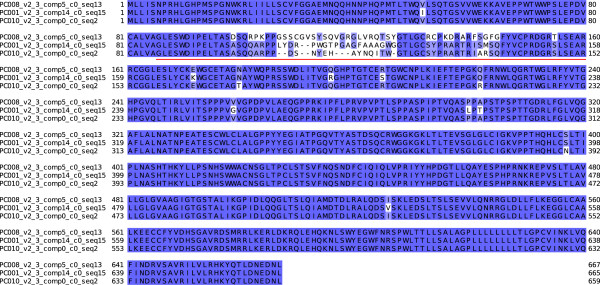
**Alignment of three PC KoRV** ***env*** **protein sequences.** The red line marks variable region A.

A second group of *env* sequences (aligned in Additional file 11) is most closely related to the published KoRV-A *env* sequence with database accession [GenBank:BAN63360] [32]. PC has a single sequence with 20 amino acid differences from the published sequence and Bi a single sequence with 23 differences. The Bi and PC sequences are 97% identical (17 amino acid changes over 665). Although BAN63360 has a high level (94%) of sequence identity to the published exemplar of the first group (BAM67147) we nevertheless consider it categorically distinct because of the necessity for introducing gaps into the alignment at three positions, and also because of the concentration of amino acid changes at positions 99-145 which is within variable region A (Figure 4).

The third group (Additional file 12) comprises a single sequence found in PC only which is most closely related to the published sequence with database accession [GenBank:AGO86848] [25]. In contrast to the published sequences mentioned above, [GenBank:AGO86848] is a KoRV-B sequence. There are no KoRV-B-like *env* sequences in the Bi transcriptome. To check this we aligned Bi reads to a KoRV-B reference sequence. Although reads align to regions conserved in KoRV-A and KoRV-B there are no alignments in the KoRV-B variable region A (result not shown). In summary there is a KoRV-B-like *env* gene expressed in PC but not in Bi. PC-derived sequences representing each of the three groups are aligned in Figure 4.

## Discussion

### Transcriptome resource

Our transcriptome dataset covering approximately 15,000 koala genes is a valuable resource for koala genetic studies and evolutionary studies of marsupials and other vertebrates. The transcript sequences have been quantified, annotated, and made available for browsing and searching at http://koalagenome.org. We envisage several uses of the dataset, some of which can be summarised as follows. Firstly, it is straightforward to get the sequences of all koala transcripts relevant to a field of interest. As an example, characterisation of the immune capability of the koala can inform prophylactic and therapeutic strategies but despite interest in this area there has as yet been only a handful of genes that have been identified and characterised. The expression of the koala interferon gamma gene has been investigated [39] because of its key role in the immune response to chlamydial infection. A more recent study [73] identified and studied the expression profiles of interferon gamma and several other koala immune response genes. Our transcriptome resource now enables the identification of a much larger number of koala immune response genes. Secondly, the dataset can used to help improve gene annotation of other marsupial genomes. We note that many of our sequences align to non-annotated regions of the opossum and Tasmanian devil genomes (not shown). Some may correspond to genes which are as yet unannotated by Ensembl. Thirdly, although there are only two individuals in this study, given their geographic separation, it should be possible to generate candidate SNPs for use in population studies. Fourthly, since we have studied a male and a female it may be possible to investigate gender-specific gene expression. Finally, the transcriptome will also aid with assembly and annotation of the koala genome, a project that is in progress.

### KoRV

This work is the most detailed report to date of KoRV transcription, and the first report of variation in KoRV expression levels in different tissues. We have analysed the KoRV transcriptional organisation and detailed its sequence variation, including the observation of a KoRV-B sequence. To the best of our knowledge this is the first report of a KoRV-B sequence in a wild population.

#### Transcriptional organization

The genetic organisation of gamma retroviruses, including KoRV and MMLV, is well understood [68, 74]. Transcription proceeds from a promoter in the 5′ LTR and proceeds through the viral genes to the 3′ LTR which contains a polyadenlylation signal. This polygenic transcript is used to express the *gag-pol* polyprotein. The virus usually also produces a spliced transcript which contains the *env* gene only. Our analysis of KoRV transcripts confirms that an *env* is spliced in accordance with the standard model, with splice sites very similar to those in MMLV, a well-characterised gamma retrovirus. Although our data also include non-canonical KoRV transcripts (unspliced portions of the genome) these are likely to be the result of assembly artefacts.

#### KoRV-B-like sequences

We approached the assessment of KoRV sequence variation in two ways: by calling SNPs and indels from reads aligned to a KoRV reference sequence; and by examining assembled transcripts. Both approaches indicate that there is a higher level of sequence diversity in PC than in Bi. Both individuals have a variety of KoRV-A sequences which is consistent with earlier reports of KoRV-A *env* sequence variation within an individual [27, 30, 32, 35]. In addition, the PC transcriptome includes an *env* sequence which appears to belong to the KoRV-B subtype. Animals from the Los Angeles Zoo studied by Xu et al. [25] were positive for both KoRV-A and KoRV-B, with the mode of spread of KoRV-B consistent with that of an exogenous virus. A possibility is that while PC and Bi are both positive for endogenous KoRV-A, PC has, in addition, an exogenous KoRV-B infection.

KoRV-B is thought to have emerged more recently than KoRV-A but its origin is not understood. A possibility is that it derived from recombination between KoRV-A and another, as yet, unidentified retrovirus. We searched our transcriptome data for sequences that might provide a clue as to the origin of KoRV-B. We note that some Velvet-Oases transcripts are similar to the possum gamma retrovirus sequence with database accession [GenBank:AF224725] [75] (alignments not shown). However, this virus does not seem to be complete and is most likely an endogenised retrovirus incapable of infection, and the sequences are quite distinct from the KoRV sequences.

Xu et al. [25] suggest that KoRV-B is linked with the development of neoplastic disease and propose that assessment of the KoRV-B status of animals should become part of the management of captive populations. The KoRV-B-like sequences we have assembled, together with the KoRV SNP data, should be useful in the development of sequence-based screening tests.

#### Assembly artefacts

Assembly of KoRV transcripts proved challenging. Our Velvet-Oases assembly procedure produced a number of KoRV transcripts but three observations suggest that they needed improvement. Firstly, some of the KoRV transcript sequences are derived from Velvet-Oases loci that have been partitioned into thousands of transcripts. Although this number was subsequently greatly reduced (in the cd-hit step to remove redundant sequences) it shows that reconstruction of KoRV transcripts is categorically different from reconstruction of most other transcripts, whose Velvet-Oases loci are not partitioned to such a degree. Secondly, in no case is the organisation of KoRV transcripts consistent with the classic transcriptional organisation of gamma retrovirus genomes. There is considerable variation in the makeup of these transcripts: the amount of the KoRV genome (which is 8.4 kb in total) present varies from fragments as small as 40 bp to near full-length. In many cases parts of the KoRV genome are repeated or rearranged and the LTR is placed centrally rather than at an end. Thirdly, some transcripts also contain reading frame errors which, on inspection of read alignments to KoRV reference sequence, are not supported (analysis not shown). Together, these observations suggested to us that in this particular set of transcripts i) the Velvet-Oases merging step is introducing errors and ii) the repetitious nature of the virus is confounding assembly. There are two sources of KoRV repeats: firstly, KoRV, like other retroviruses, contains a long terminal repeat (LTR) sequence (the KoRV-A LTR is 505 bp); and secondly, the integrated virus is present at multiple loci in the genome (viral copy number has been estimated at up to 200 copies per cell [28]). Reassembly with non-LTR reads seemed to mitigate the problem since we were able to produce reconstructed KoRV transcripts with the expected structure. We have confidence in the KoRV sequences reported here since i) in some cases the sequences match exactly with previously published data; and ii) the assembly is supported by alignments of sequence reads back to the transcripts (analysis not shown). The sequencing of the genome of these two individuals will reveal the full extent of KoRV provirus sequence diversity.

## Conclusions

The transcriptome resource presented here is a useful resource for studies of genetic diversity within populations, of genes underlying disease resistance, and of the genetic basis of the unique adaptive features of the koala. The results increase our knowledge of KoRV transcription and of the extent of KoRV sequence variation.

## Availability of supporting data

### Accession numbers

Sequence reads have been deposited with the NCBI Sequence Read Archive (SRA) and have received accessions SRR1203868, SRR1205138, SRR1205176, SRR1205998, SRR1205218, SRR1205223, SRR1205222 and SRR1205224 (PC); and SRR1106690, SRR1106707, SRR1121764, SRR1122141, SRR1207973, SRR1207974 and SRR1207975 (Bi).

Assembled sequences can be queried and browsed at the koala genome website [63]. Alignments of koala transcripts to the opossum and Tasmanian devil genomes are available as DAS sources summarised on the koalagenome.org DSN page [76].

## Authors’ information

The authors are involved in the Koala Genome Consortium [63].

These authors Rebecca N Johnson and Peter Timms are joint last authors of this work.

## Electronic supplementary material

Additional file 1: **Description of two pilot transcriptome assemblies.** (PDF 98 KB)

Additional file 2: **Summary of protein BLAST sequence alignments of koala proteins to proteins from Ensembl annotations of three marsupial genomes (Tasmanian devil, opossum and tammar wallaby) with RBH constraint applied.** (XLSX 7 MB)

Additional file 3: **Transcript abundance.** (XLS 566 KB)

Additional file 4: **Differentially expressed transcripts.** For each library the top 100 transcripts, ranked by the logFR value produced by edgeR, are listed. (XLS 877 KB)

Additional file 5: **Alignment of koala alpha amylase sequences.** (PDF 27 KB)

Additional file 6: **Alignment of koala aldehyde reductase sequences.** (PDF 20 KB)

Additional file 7: **SNPs and small indels within KoRV genome.** (XLSX 25 KB)

Additional file 8: **Alignment of KoRV** ***gag*** **protein sequences.** The first part of the sequence identifier can be used to infer the name of the library from which the sequence was obtained. (PDF 18 KB)

Additional file 9: **Alignment of KoRV** ***pol*** **protein sequences.** (PDF 24 KB)

Additional file 10: **Alignment of PC and Bi KoRV** ***env*** **protein sequences related to the KoRV-A sequence BAM67147.** Two of the sequences (from PC library PC010 and Bi lymph node library) are identical to [GenBank:BAM67147]. (PDF 19 KB)

Additional file 11: **Alignment of PC and Bi KoRV** ***env*** **protein sequences with the KoRV-A sequence [GenBank:BAN63360].** (PDF 20 KB)

Additional file 12: **Alignment of a PC KoRV** ***env*** **protein sequence with the KoRV-B sequence [GenBank:AGO86848].** (PDF 18 KB)

## Abbreviations

PC: Pacific Chocolate
Bi: Birke
KoRV: koala retrovirus
mya: Million years ago
FPKM: Fragments per kilobase of exon per million fragments mapped
Bp: Base pairs
kb: Kilobase pairs
Mb: Megabase pairs
Gb: Gigabase pairs.
