# Supplementary material for: A transcriptome resource for the koala (Phascolarctos cinereus): insights into koala retrovirus transcription and sequence diversity
Source: BMC Genomics. 2014 Sep 11;15(1):786. doi: 10.1186/1471-2164-15-786 (PMC4247155; doi:10.1186/1471-2164-15-786)
Supplement: Supplementary file 1 — Additional file 1: Description of two pilot transcriptome assemblies. (PDF 98 KB) [file 12864_2014_6686_MOESM1_ESM.pdf]

Comparison of assembly with Velvet-Oases and Trinity

In a first set of pilot assemblies we investigated two different assembly methods (Velvet-Oases and Trinity), each of which used sequence data from a single PC library as input. The Velvet-Oases method was run on eight libraries while Trinity was run on three only. Table S1 shows several descriptive metrics of assembly, including number of transcripts and N50. For Velvet-Oases assemblies the number of transcripts ranged from 87,666 (PC009) to 210,518 (PC001), and N50 ranged from 2,513 (PC004) to 4,161 (PC010). Although these values are not necessarily good indicators of the quality of a transcriptome assembly [R1], we nevertheless note that they are consistent with naive expectations for a marsupial transcriptome. For example, in the Ensembl gene annotation of the opossum, a marsupial with a well-annotated genome (Table 1 in main text), the mean length of 22,310 predicted protein-coding transcripts is 2,595. For the three libraries treated with both assembly methods, Trinity gave similar results to the Velvet-Oases assemblies, although Velvet-Oases produced fewer transcripts and higher N50 values.

We aimed to construct an extensive catalog of koala transcripts and so the number of genes represented is a more pertinent measure of the quality of transcriptome assembly than is N50. Since there is no koala reference genome or transcriptome available for this purpose we used instead sequences from the Tasmanian devil. We used BLAST to align koala transcript sequences to protein products of Tasmanian devil Ensembl genes (Table S1). In the top-scoring alignments from searches with Velvet-Oases transcripts there are between 11,973 (in PC009) and 14,508 (PC008) distinct Tasmanian devil protein sequences, with the corresponding number of genes ranging between 10,959 and 13,108. This rough estimate of

gene coverage indicates that the Velvet-Oases assemblies are successfully reconstructing transcripts from a large number of genes. Analogous BLAST searches with the three sets of transcripts produced by Trinity give comparable numbers of proteins and genes, although in two libraries (PC001 and PC005) these numbers are higher than for Velvet-Oases. However, this relationship is not constant when we take into consideration the length of the alignment. Figure S1 shows the number of distinct Tasmanian devil protein sequences in Velvet-Oases and Trinity PC001 library alignments when various values of an alignment length filter (minimum proportion of subject sequence covered by the alignment) are applied. When this length requirement is 20% or more the number of proteins is higher in Velvet-Oases than in Trinity. A similar situation is seen in the case of PC005 alignments (not shown). This suggests that for our data Velvet-Oases is slightly better than Trinity at reconstructing intact full-length protein coding regions. We conclude that both Velvet-Oases and Trinity give satisfactory and comparable results, but chose to proceed with Velvet-Oases in subsequent assemblies because of its apparent advantage in producing transcripts encoding complete protein sequences.

Effect on assembly of digital normalization of input data

In a second set of assemblies, for three of the PC libraries (as before, PC001, PC005 and PC006) we investigated the effect on Velvet-Oases assembly outcomes of reducing the size of the input data with a digital normalization procedure (which we planned to use on a global assembly). The results (Table S1) show that in comparison with Velvet-Oases assemblies of non-normalized data there were reductions in the number of transcripts, in N50 and in gene coverage again as measured by the number of distinct Tasmanian devil genes represented in

sequence alignments. The size of the reductions varies between libraries, with PC001 being least affected (e.g. the number of Tasmanian devil genes covered in BLAST searching is reduced by only 0.8% to 12,538), while in PC006 the reductions are quite large (e.g. the number of Tasmanian devil genes covered in BLAST searching is reduced by 35% (from 11,707 to to 7,585). We conclude that the normalization is effective but is causing loss of information which is particularly noticeable in PC006. Nevertheless when scaling up the assembly process this potential loss is outweighed by the advantage offered by reduction in computational requirements (because size of input to the assembly program is reduced) and so we used this method to construct our final global (all tissue libraries) assemblies for PC and for Bi.

Table S1 Koala pilot transcriptome assemblies.

Library	Method	No. transcripts	Max. length	Mean length	N50	No. proteins hit[1]	No. genes[2]
PC001	Trinity	242163	22842	1208	3165	14435	13334
	Velvet-Oases	210518	23408	1851	3424	14065	12651
	digital normalization Velvet-Oases	133329	22850	1741	3139	13892	12538
PC004	Velvet-Oases	100884	26585	1485	2513	12012	10960
PC005	Trinity	138927	22777	1140	2796	13130	12225
	Velvet-Oases	118557	22704	1904	3291	13141	11995
	digital normalization Velvet-Oases	89660	21425	1645	2886	12838	11911
PC006	Trinity	157396	18583	1103	2769	11624	10837
	Velvet-Oases	122763	22043	1868	3203	12830	11707
	digital normalization Velvet-Oases	42624	12711	978	1604	8086	7585
PC008	Velvet-Oases	180514	23671	2050	3642	14508	13108
PC009	Velvet-Oases	87666	89224	1689	3042	11973	10959
PC010	Velvet-Oases	147527	31170	2298	4161	14204	12994
PC011	Velvet-Oases	124433	22391	2136	3754	13367	12183

1. Total number of distinct protein sequences in best-hit translated BLAST alignments of koala transcripts with Ensembl Tasmanian devil proteins.
2. Total number of distinct Tasmanian devil genes.

Figure S1

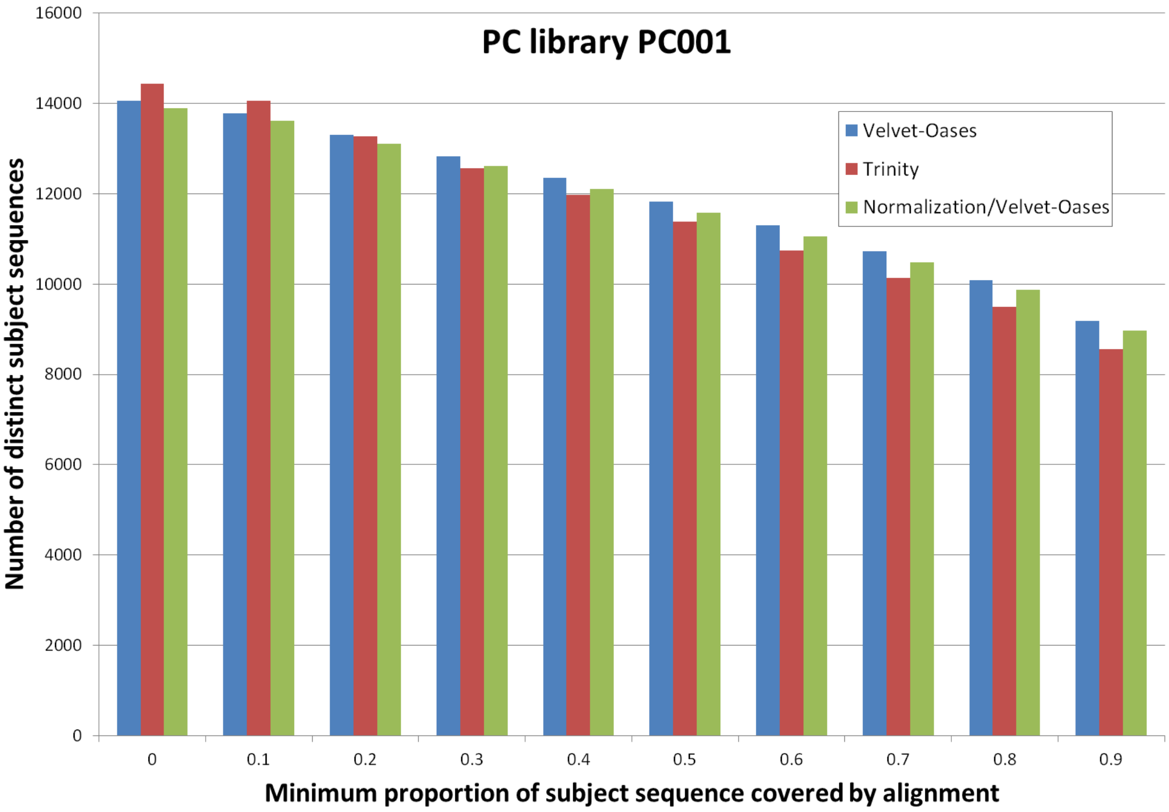

Number of protein sequences found by translated BLAST similarity searching with transcripts produced by three transcriptome assembly procedures. Koala transcript sequences from assemblies of a representative library (PC_0001; spleen) were used to query Tasmanian devil protein sequences and the top hit was retained. The number of distinct subject sequences is shown for a range of minimum alignment length constraints.

References

R1. Baker M: **De novo genome assembly: what every biologist should know**. *Nat Meth* 2012, 9(4):333-337.
