# Supplementary material for: A transcriptome resource for the koala (Phascolarctos cinereus): insights into koala retrovirus transcription and sequence diversity
Source: BMC Genomics. 2014 Sep 11;15(1):786. doi: 10.1186/1471-2164-15-786 (PMC4247155; doi:10.1186/1471-2164-15-786)
Supplement: Supplementary file 5 — Additional file 5: Alignment of koala alpha amylase sequences. (PDF 27 KB) [file 12864_2014_6686_MOESM5_ESM.pdf]

```
m 71048      1 MKFLLLLSVLGVCWAQYDPHTKYGRTSIVHLFEWRWDDIALECERYLAPNGFGGVQVSPPNENAVINNPSRPWWERYQPI 80
m 71055      1 MKFLLLLSVLGVCWAQYDPHTKYGRTSIVHLFEWRWDDIALECERYLAPNGFGGVQVSPPNENAVINNPSRPWWERYQPI 80
m 71059      1 MRLLLWLSVLGVCWAQYDPHTKYGRTSIVHLFEWRWDDIALECERYLAPNGFGGVQVSPPNENAVINNPSRPWWERYQPI 80
m 71064      1 MRLLLWLSVLGVCWAQYDPHTKYGRTSIVHLFEWRWDDIALECERYLAPNGFGGVQVSPPNENAVINNPSRPWWERYQPI 80
m 71067      1 MKFLLLLSVLGVCWAQYDPHTKYGRTSIVHLFEWRWDDIALECERYLAPNGFGGVQVSPPNENAVINNPSRPWWERYQPI 80
m 71077      1 MKFLLLLSVLGVCWAQYDPHTKYGRTSIVHLFEWRWDDIALECERYLAPNGFGGVQVSPPNENAVINNPSRPWWERYQPI 80
m 71094      1 MKFLLLLSVLGVCWAQYDPHTKYGRTSIVHLFEWRWDDIALECERYLAPNGFGGVQVSPPNENAVINNPSRPWWERYQPI 80
m 71097      1 MKFLLLLSVLGVCWAQYDPHTKYGRTSIVHLFEWRWDDIALECERYLAPNGFGGVQVSPPNENAVINNPSRPWWERYQPI 80
m 71026      1 MKFLLLLSVLGVCWAQYDPHTKYGRTSIVHLFEWRWDDIALECERYLAPNGFGGVQVSPPNENAVINNPSRPWWERYQPI 80
qm 155617    1 MRLLLWLSVLGVCWAQYNPHTQYGRTSIVHLFEWRWDDIALECERYLAPNGFGGVQVSPPNENAVINNPSRPWWERYQPI 80
qm 155619    1 MRLLLWLSVLGVCWAQYNPHTQYGRTSIVHLFEWRWDDIALECERYLAPNGFGGVQVSPPNENAVINNPSRPWWERYQPI 80
qm 155623    1 MRLLLWLSVLGVCWAQYNPHTQYGRTSIVHLFEWRWDDIALECERYLAPNGFGGVQVSPPNENAVINNPSRPWWERYQPI 80
qm 155628    1 MRLLLWLSVLGVCWAQYNPHTQYGRTSIVHLFEWRWDDIALECERYLAPNGFGGVQVSPPNENAVINNPSRPWWERYQPI 80
qm 155630    1 MKFLLLLSVLGVCWAQYDPHTKYGRTSIVHLFEWRWDDIALECERYLAPNGYGGVQVSPPNENAVINNPSRPWWERYQPI 80
qm 155640    1 MKFLLLLSVLGVCWAQYDPHTKYGRTSIVHLFEWRWDDIALECERYLAPNGFGGVQVSPPNENAVINNPSRPWWERYQPI 80
qm 155666    1 MKFLLLLSVLGVCWAQYDPHTKYGRTSIVHLFEWRWDDIALECERYLAPNGYGGVQVSPPNENAVINNPSRPWWERYQPI 80
qm 155688    1 MKFLLLLSVLGVCWAQYDPHTKYGRTSIVHLFEWRWDDIALECERYLAPNGFGGVQVSPPNENAVINNPSRPWWERYQPI 80
qm 155691    1 MKFLLLLSVLGVCWAQYDPHTKYGRTSIVHLFEWRWDDIALECERYLAPNGFGGVQVSPPNENAVINNPSRPWWERYQPI 80
qm 155697    1 MKFLLLLSVLGVCWAQYDPHTKYGRTSIVHLFEWRWDDIALECERYLAPNGYGGVQVSPPNENAVINNPSRPWWERYQPI 80

m 71048     81 SYKICSRSGNEDEFRDMVKRCNDVGVNIYVDAVVNHMCGSGLSAGTSSTCGSYYNPGTREFSGVPYSAWDFNDGKCQTSS 160
m 71055     81 SYKICSRSGNEDEFRDMVKRCNDVGVNIYVDAVVNHMCGSGLSAGTSSTCGSYYNPGTREFSGVPYSAWDFNDGKCQTSS 160
m 71059     81 SYKICSRSGNEDEFRDMVKRCNDVGVNIYVDAVVNHMCGSGLSAGTSSTCGSYYNPGTRDFPAVPYSGWDFNDGKCNTRS 160
m 71064     81 SYKICSRSGNEDEFRDMVKRCNDVGVNIYVDAVVNHMCGSGLSAGTSSTCGSYYNPGTREFSGVPYSAWDFNDGKCQTSS 160
m 71067     81 SYKLCTRSGSEDDFIDMVQRCNNVGVNIYVDAVVNHMCGSGLSAGTSSTCGSYYNPGTREFSGVPYSAWDFNDGKCQTSS 160
m 71077     81 SYKICSRSGNEDEFRDMVKRCNDVGVNIYVDAVVNHMCGSGLSAGTSSTCGSYYNPGTRDFPAVPYSGWDFNDGKCNTRS 160
m 71094     81 SYKLCTRSGSEDDFIDMVQRCNNVGVNIYVDAVVNHMCGSGLSAGTSSTCGSYYNPGTREFSGVPYSAWDFNDGKCQTSS 160
m 71097     81 SYKICSRSGNEDEFRDMVKRCNDVGVNIYVDAVVNHMCGSGLSAGTSSTCGSYYNPGTRDFPAVPYSGWDFNDGKCNTRS 160
m 71026     81 SYKLCTRSGSEDDFIDMVQRCNNVGVNIYVDAVVNHMCGSGLSAGTSSTCGSYYNPGTRDFPAVPYSGWDFNDGKCNTRS 160
qm 155617   81 SYKMCSRSGNEDEFRDMVKRCNDVGVNIYVDAVVNHMCGSGVSAGTSSTCGSYYNPGTRDFPAVPYSGWDFNDGKCNTRS 160
qm 155619   81 SYKMCSRSGNEDEFRDMVKRCNDVGVNIYVDAVVNHMCGSGVSAGTSSTCGSYYNPGTRDFPAVPYSGWDFNDGKCNTRS 160
qm 155623   81 SYKMCSRSGNEDEFRDMVKRCNDVGVNIYVDAVVNHMCGSGVSAGTSSTCGSYYNPGTRDFPAVPYSGWDFNDGKCNTRS 160
qm 155628   81 SYKMCSRSGNEDEFRDMVKRCNDVGVNIYVDAVVNHMCGSGVSAGTSSTCGSYYNPGTRDFPAVPYSGWDFNDGKCNTRS 160
qm 155630   81 SYKLCTRSGSEDDFIDMVQRCNNVGVNIYVDAVVNHMCGSGLSAGTSSTCGSYYNPGTREFSGVPYSAWDFNDGKCQTSS 160
qm 155640   81 SYKMCSRSGNEDEFRDMVKRCNDVGVNIYVDAVVNHMCGSGVSAGTSSTCGSYYNPGTRDFPAVPYSGWDFNDGKCNTRS 160
qm 155666   81 SYKLCTRSGSEDDFIDMVQRCNNVGVNIYVDAVVNHMCGSGVSAGTSSTCGSYYNPGTRDFPAVPYSGWDFNDGKCNTRS 160
qm 155688   81 SYKMCSRSGNEDEFRDMVKRCNDVGVNIYVDAVVNHMCGSGVSAGTSSTCGSYYNPGTRDFPAVPYSGWDFNDGKCNTRS 160
qm 155691   81 SYKMCSRSGNEDEFRDMVKRCNDVGVNIYVDAVVNHMCGSGVSAGTSSTCGSYYNPGTRDFPAVPYSGWDFNDGKCNTRS 160
qm 155697   81 SYKLCTRSGSEDDFIDMVQRCNNVGVNIYVDAVVNHMCGSGLSAGTSSTCGSYYNPGTREFSGVPYSAWDFNDGKCQTSS 160

m 71048    161 GEIESYYDIYQVRDCRLSGLLDLAQENDYVRQMIAAYLNHLIGIGVAGFRIDASKHMWPGDMKAFLDKLHNLNITWFPQG 240
m 71055    161 GEIESYYDIYQVRDCRLSGLLDLAQENDYVRQMIAAYLNHLIGIGVAGFRIDASKHMWPGDMKAFLDKLHNLNITWFPQG 240
m 71059    161 GEIESYSDIYQVRDCRLFGLLDLALEKDYVRSMIASYLNHLIDMGVAGFRIDASKHMWPGDMKAFLDKLHNLNITWFPQG 240
m 71064    161 GEIESYYDIYQVRDCRLSGLLDLAQENDYVRQMIAAYLNHLIGIGVAGFRIDASKHMWPGDMKAFLDKLHNLNITWFPQG 240
m 71067    161 GEIESYYDIYQVRDCRLSGLLDLAQENDYVRQMIAAYLNHLIGIGVAGFRIDASKHMWPGDIKAILDQLNNLNTNWFPSG 240
m 71077    161 GEIESYSDIYQVRDCRLFGLLDLALEKDYVRSMIASYLNHLIDMGVAGFRIDASKHMWPGDMKAFLDKLHNLNITWFPQG 240
m 71094    161 GEIESYYDIYQVRDCRLSGLLDLAQENDYVRQMIAAYLNHLIGIGVAGFRIDASKHMWPGDIKAILDQLNNLNTNWFPSG 240
m 71097    161 GEIESYSDIYQVRDCRLFGLLDLALEKDYVRSMIASYLNHLIDMGVAGFRIDASKHMWPGDMKAFLDKLHNLNITWFPQG 240
m 71026    161 GEIESYSDIYQVRDCRLFGLLDLALEKDYVRSMIASYLNHLIDMGVAGFRIDASKHMWPGDMKAFLDKLHNLNITWFPQG 240
qm 155617  161 GEIESYSDIYQVRDCRLFGLLDLALEKDYVRSMIASYLNHLIDMGVAGFRIDASKHMWPGDMKAFLDKLHNLNITWFPQG 240
qm 155619  161 GEIESYSDIYQVRDCRLFGLLDLALEKDYVRSMIASYLNHLIDMGVAGFRIDASKHMWPGDMKAFLDKLHNLNITWFPQG 240
qm 155623  161 GEIESYSDIYQVRDCRLFGLLDLALEKDYVRSMIASYLNHLIDMGVAGFRIDASKHMWPGDMKAFLDKLHNLNITWFPQG 240
qm 155628  161 GEIESYSDIYQVRDCRLFGLLDLALEKDYVRSMIASYLNHLIDMGVAGFRIDASKHMWPGDMKAFLDKLHNLNITWFPQG 240
qm 155630  161 GEIESYYDIYQVRDCRLSGLLDLAQENDYVRQMIAAYLNHLIGIGVAGFRIDASKHMWPGDIKAILDQLNNLNTNWFPSG 240
qm 155640  161 GEIESYSDIYQVRDCRLFGLLDLALEKDYVRSMIASYLNHLIDMGVAGFRIDASKHMWPGDMKAFLDKLHNLNITWFPQG 240
qm 155666  161 GEIESYSDIYQVRDCRLFGLLDLALEKDYVRSMIASYLNHLIDMGVAGFRIDASKHMWPGDMKAFLDKLHNLNITWFPQG 240
qm 155688  161 GEIESYSDIYQVRDCRLFGLLDLALEKDYVRSMIASYLNHLIDMGVAGFRIDASKHMWPGDMKAFLDKLHNLNITWFPQG 240
qm 155691  161 GEIESYSDIYQVRDCRLFGLLDLALEKDYVRSMIASYLNHLIDMGVAGFRIDASKHMWPGDMKAFLDKLHNLNITWFPQG 240
qm 155697  161 GEIESYYDIYQVRDCRLSGLLDLAQENDYVRQMIAAYLNHLIGIGVAGFRIDASKHMWPGDIKAILDQLNNLNTNWFPSG 240

m 71048    241 ARPFIYQEVIDLVGEPIKSSEYFGNGRVTEFKFGAKLGTVVRKWNGEKMAYLKNWGEGWAFMPSDRALVFVDNHDNQRGH 320
m 71055    241 ARPFIYQEVIDLVGEPIKSSEYFGNGRVTEFKFGAKLGTVVRKWNGEKMAYLKNWGEGWAFMPSDRALVFVDNHDNQRGH 320
m 71059    241 ARPFIYQEVIDLVGEPIKSSEYFGNGRVTEFKFGAKLGTVVRKWNGEKMAYLKNWGEGWAFMPSDRALVFVDNHDNQRGH 320
m 71064    241 ARPFIYQEVIDLVGEPIKSSEYFGNGRVTEFKFGAKLGTVVRKWNGEKMAYLKNWGEGWAFMPSDRALVFVDNHDNQRGH 320
m 71067    241 SRPFIYQEVIDLGNEPITSSEYFDNGRVTEFKYGAKLGTVIRKWDGEMMTYLSNWGEGWSFVRSDRALVFVDNHDNQRGH 320
m 71077    241 ARPFIYQEVIDLVGEPIKSSEYFGNGRVTEFKFGAKLGTVVRKWNGEKMAYLKNWGEGWAFMPSDRALVFVDNHDNQRGH 320
m 71094    241 SRPFIYQEVIDLGNEPITSSEYFDNGRVTEFKYGAKLGTVIRKWDGEMMTYLSNWGEGWSFVRSDRALVFVDNHDNQRGH 320
m 71097    241 ARPFIYQEVIDLVGEPIKSSEYFGNGRVTEFKFGAKLGTVVRKWNGEKMAYLKNWGEGWAFMPSDRALVFVDNHDNQRGH 320
m 71026    241 ARPFIYQEVIDLVGEPIKSSEYFGNGRVTEFKFGAKLGTVVRKWNGEKMAYLKNWGEGWAFMPSDRALVFVDNHDNQRGH 320
qm 155617  241 ARPFIYQEVIDLGGETIKSSEYFGNGRVTEFKFGAKLGTVVRKWNGEKMAYLKNWGEGWAFMPSDRALVFVDNHDNQRGH 320
qm 155619  241 ARPFIYQEVIDLGGETIKSSEYFGNGRVTEFKFGAKLGTVVRKWNGEKMAYLKNWGEGWAFMPSDRALVFVDNHDNQRGH 320
qm 155623  241 ARPFIYQEVIDLGGETIKSSEYFGNGRVTEFKFGAKLGTVVRKWNGEKMAYLKNWGEGWAFMPSDRALVFVDNHDNQRGH 320
qm 155628  241 ARPFIYQEVIDLGGETIKSSEYFGNGRVTEFKFGAKLGTVVRKWNGEKMAYLKNWGEGWAFMPSDRALVFVDNHDNQRGH 320
qm 155630  241 SRPFIYQEVIDLGNEPITSSEYFDNGRVTEFKYGAKLGTVIRKWDGEMMTYLSNWGEGWSFVRSDRALVFVDNHDNQRGH 320
qm 155640  241 ARPFIYQEVIDLGGETIKSSEYFGNGRVTEFKFGAKLGTVVRKWNGEKMAYLKNWGEGWAFMPSDRALVFVDNHDNQRGH 320
qm 155666  241 ARPFIYQEVIDLGGETIKSSEYFGNGRVTEFKFGAKLGTVVRKWNGEKMAYLKNWGEGWAFMPSDRALVFVDNHDNQRGH 320
qm 155688  241 ARPFIYQEVIDLGGETIKSSEYFGNGRVTEFKFGAKLGTVVRKWNGEKMAYLKNWGEGWAFMPSDRALVFVDNHDNQRGH 320
qm 155691  241 ARPFIYQEVIDLGGETIKSSEYFGNGRVTEFKFGAKLGTVVRKWNGEKMAYLKNWGEGWAFMPSDRALVFVDNHDNQRGH 320
qm 155697  241 SRPFIYQEVIDLGNEPITSSEYFDNGRVTEFKYGAKLGTVIRKWDGEMMTYLSNWGEGWSFVRSDRALVFVDNHDNQRGH 320

m 71048    321 GAGGDSILTFWDARLYKMAVGFMLAHPYGFTRVMSSFSWPRNFQNGQDSNDWVGPPNDNGVIKRVTINEDTTCGNDWVCE 400
m 71055    321 GAGGDSILTFWDARLYKMAVGFMLAHPYGFTRVMSSFSWPRNFQNGQDSNDWVGPPNDNGVIKRVTINEDTTCGNDWVCE 400
m 71059    321 GAGGDSILTFWDARLYKMAVGFMLAHPYGFTRVMSSFSWPRNFQNGQDSNDWVGPPNDNGVIKRVTINEDTTCGNDWVCE 400
m 71064    321 GAGGDSILTFWDARLYKMAVGFMLAHPYGFTRVMSSFSWPRNFQNGQDSNDWVGPPNDNGVIKRVTINEDTTCGNDWVCE 400
m 71067    321 GAGGDSILTFWDARLYKMAVGFMLAHPYGFTRVMSSFSWPRNFQNGQDSNDWVGPPNDNGVIKRVTINEDTTCGNDWVCE 400
m 71077    321 GAGGDSILTFWDARLYKMAVGFMLAHPYGFTRVMSSFSWPRNFQNGQDSNDWVGPPNDNGVIKRVTINEDTTCGNDWVCE 400
m 71094    321 GAGGDSILTFWDARLYKMAVGFMLAHPYGFTRVMSSFSWPRNFQNGQDSNDWVGPPNDNGVIKRVTINEDTTCGNDWVCE 400
m 71097    321 GAGGDSILTFWDARLYKMAVGFMLAHPYGFTRVMSSFSWPRNFQNGQDSNDWVGPPNDNGVIKRVTINEDTTCGNDWVCE 400
m 71026    321 GAGGDSILTFWDARLYKMAVGFMLAHPYGFTRVMSSFSWPRNFQNGQDSNDWVGPPNDNGVIKRVTINEDTTCGNDWVCE 400
qm 155617  321 GAGGASILTFWDSRMYKMAVGFMLAHPYGFTRVMSSFSWPRNFQNGQDSNDWVGPPNDNGVIKRVTINEDTTCGNDWVCE 400
qm 155619  321 GAGGASILTFWDSRMYKMAVGFMLAHPYGFTRVMSSFSWPRNFQNGQDSNDWVGPPNDNGVIKRVTINEDTTCGNDWVCE 400
qm 155623  321 GAGGDSILTFWDARLYKMAVGFMLAHPYGFTRVMSSFSWPRNFQNGQDSNDWVGPPNDNGVIKRVTINEDTTCGNDWVCE 400
qm 155628  321 GAGGDSILTFWDARLYKMAVGFMLAHPYGFTRVMSSFSWPRNFQNGQDSNDWVGPPNDNGVIKRVTINEDTTCGNDWVCE 400
qm 155630  321 GAGGASILTFWDSRMYKMAVGFMLAHPYGFTRVMSSYRWSRNIQNGEDTNDWIGPPNDNGVIKRVTINEDTTCGNDWVCE 400
qm 155640  321 GAGGDSILTFWDARLYKMAVGFMLAHPYGFTRVMSSFSWPRNFQNGQDSNDWVGPPNDNGVIKRVTINEDTTCGNDWVCE 400
qm 155666  321 GAGGASILTFWDSRMYKMAVGFMLAHPYGFTRVMSSFSWPRNFQNGQDSNDWVGPPNDNGVIKRVTINEDTTCGNDWVCE 400
qm 155688  321 GAGGDSILTFWDARLYKMAVGFMLAHPYGFTRVMSSFSWPRNFQNGQDSNDWVGPPNDNGVIKRVTINEDTTCGNDWVCE 400
qm 155691  321 GAGGDSILTFWDARLYKMAVGFMLAHPYGFTRVMSSYRWSRNIQNGEDTNDWIGPPNDNGVIKRVTINEDTTCGNDWVCE 400
qm 155697  321 GAGGASILTFWDSRMYKMAVGFMLAHPYGFTRVMSSFSWPRNFQNGQDSNDWVGPPNDNGVIKRVTINEDTTCGNDWVCE 400

m 71048    401 HRWRQIRNMVIFRNVVNGQDFTNWWDNGSNQVAFGRGNKGFIVFNNDDWALLSNLQTGLPAGTYCDVISGDKSDNSCTGI 480
m 71055    401 HRWRQIRNMVIFRNVVNGQDFTNWWDNGSNQVAFGRGNKGFIVFNNDDWALLSNLQTGLPAGTYCDVISGDKIHKRCTGI 480
m 71059    401 HRWRQIRNMVIFRNVVNGQDFTNWWDNGSNQVAFGRGNKGFIVFNNDDWALLSNLQTGLPAGTYCDVISGDKIHKRCTGI 480
m 71064    401 HRWRQIRNMVIFRNVVNGQDFTNWWDNGSNQVAFGRGNKGFIVFNNDDWALLSNLQTGLPAGTYCDVISGDKIHKRCTGI 480
m 71067    401 HRWRQIRNMVIFRNVVNGQDFTNWWDNGSNQVAFGRGNKGFIVFNNDDWALLSNLQTGLPAGTYCDVISGDKSDNSCTGI 480
m 71077    401 HRWRQIRNMVIFRNVVNGQDFTNWWDNGSNQVAFGRGNKGFIVFNNDDWALLSNLQTGLPAGTYCDVISGDKIHKRCTGI 480
m 71094    401 HRWRQIRNMVIFRNVVNGQDFTNWWDNGSNQVAFGRGNKGFIVFNNDDWALLSNLQTGLPAGTYCDVISGDKIHKRCTGI 480
m 71097    401 HRWRQIRNMVIFRNVVNGQDFTNWWDNGSNQVAFGRGNKGFIVFNNDDWALLSNLQTGLPAGTYCDVISGDKSDNSCTGI 480
m 71026    401 HRWRQIRNMVIFRNVVNGQDFTNWWDNGSNQVAFGRGNKGFIVFNNDDWALLSNLQTGLPAGTYCDVISGDKIHKRCTGI 480
qm 155617  401 HRWRQIRNMVIFRNVVNGEDFTNWWSNGSNQVAFGRGNKGFIVFNNDDWELSSNLQTGLPAGTYCDVISGDKSDNSCTGI 480
qm 155619  401 HRWRQIRNMVIFRNVVNGQDFTNWWDNGSNQVAFGRGNKGFIVFNNDDWALLSNLQTGLPAGTYCDVISGDKIHKRCTGI 480
qm 155623  401 HRWRQIRNMVIFRNVVNGQDFTNWWDNGSNQVAFGRGNKGFIVFNNDDWALLSNLQTGLPAGTYCDVISGDKIHKRCTGI 480
qm 155628  401 HRWRQIRNMVIFRNVVNGEDFTNWWSNGSNQVAFGRGNKGFIVFNNDDWELSSNLQTGLPAGTYCDVISGDKSDNSCTGI 480
qm 155630  401 HRWRQIRNMVIFRNVVNGEDFTNWWSNGSNQVAFGRGNKGFIVFNNDDWELSSNLQTGLPAGTYCDVISGDKSDNSCTGI 480
qm 155640  401 HRWRQIRNMVIFRNVVNGEDFTNWWSNGSNQVAFGRGNKGFIVFNNDDWELSSNLQTGLPAGTYCDVISGDKSDNSCTGI 480
qm 155666  401 HRWRQIRNMVIFRNVVNGEDFTNWWSNGSNQVAFGRGNKGFIVFNNDDWELSSNLQTGLPAGTYCDVISGDKIHKRCTGI 480
qm 155688  401 HRWRQIRNMVIFRNVVNGQDFTNWWDNGSNQVAFGRGNKGFIVFNNDDWALLSNLQTGLPAGTYCDVISGDKIHKRCTGI 480
qm 155691  401 HRWRQIRNMVIFRNVVNGEDFTNWWSNGSNQVAFGRGNKGFIVFNNDDWELSSNLQTGLPAGTYCDVISGDKSDNSCTGI 480
qm 155697  401 HRWRQIRNMVIFRNVVNGEDFTNWWSNGSNQVAFGRGNKGFIVFNNDDWELSSNLQTGLPAGTYCDVISGDKSDNSCTGI 480

m 71048    481 QVNVGSDGLAYFSISNSAEDPFIAIHVDAKL* 512
m 71055    481 EIYVGSSGLAYFSISNTAEDPFIAIHVDAKL* 512
m 71059    481 EIYVGSSGLAYFSISNTAEDPFIAIHVDAKL* 512
m 71064    481 EIYVGSSGLAYFSISNTAEDPFIAIHVDAKL* 512
m 71067    481 QVNVGSDGLAYFSISNSAEDPFIAIHVDAKL* 512
m 71077    481 EIYVGSSGLAYFSISNTAEDPFIAIHVDAKL* 512
m 71094    481 EIYVGSSGLAYFSISNTAEDPFIAIHVDAKL* 512
m 71097    481 QVNVGSDGLAYFSISNSAEDPFIAIHVDAKL* 512
m 71026    481 EIYVGSSGLAYFSISNTAEDPFIAIHVDAKL* 512
qm 155617  481 QVNVGSDGLAYFSISNSAEDPFIAIHVDAKL* 512
qm 155619  481 EIYVGSSGLAYFSISNTAEDPFIAIHVDAKL* 512
qm 155623  481 EIYVGSSGLAYFSISNTAEDPFIAIHVDAKL* 512
qm 155628  481 QVNVGSDGLAYFSISNSAEDPFIAIHVDAKL* 512
qm 155630  481 QVNVGSDGLAYFSISNSAEDPFIAIHVDAKL* 512
qm 155640  481 QVNVGSDGLAYFSISNSAEDPFIAIHVDAKL* 512
qm 155666  481 EIYVGSSGLAYFSISNTAEDPFIAIHVDAKL* 512
qm 155688  481 EIYVGSSGLAYFSISNTAEDPFIAIHVDAKL* 512
qm 155691  481 QVNVGSDGLAYFSISNSAEDPFIAIHVDAKL* 512
qm 155697  481 QVNVGSDGLAYFSISNSAEDPFIAIHVDAKL* 512
```
