# Supplementary material for: A transcriptome resource for the koala (Phascolarctos cinereus): insights into koala retrovirus transcription and sequence diversity
Source: BMC Genomics. 2014 Sep 11;15(1):786. doi: 10.1186/1471-2164-15-786 (PMC4247155; doi:10.1186/1471-2164-15-786)
Supplement: Supplementary file 6 — Additional file 6: Alignment of koala aldehyde reductase sequences. (PDF 20 KB) [file 12864_2014_6686_MOESM6_ESM.pdf]

```
qm 185823    1 MSSAARTL--PATVLGAMEMGWRMDQASSEASMQAFLDRGHREVDTAFVYGDGQSESFLGSMDLGLKDGSKGVKIATKAIP  79
m 182015     1 MSSAARTQDRPGTVLGAMEMGWRMDLAASKVCMQAFLDRGYREVDTAHYYGEGQSESFLGSMDLGLKDGSKGVKIATKAIP  81
qm 185847    1 MSSAARTQDRPGTVLGAMEMGWRMDLAASKVCMQAFLDRGYREVDTAHYYGEGQSESFLGSMDLGLKDGSKGVKIATKANP  81
qm 185832    1 MSSAARTL--PATVLGAMEMGWRMDQASSEASMQAFLDRGHREVDTAFVYGDGQSESFLGSMDLGLKDGSKGVKIATKAIP  79
m 182017     1 MSSAARTQDRPGTVLGAMEMGWRMDLAASKVCMQAFLDRGYREVDTAHYYGEGQSESFLGSMDLGLKDGSKGVKIATKAIP  81
m 182026     1 MSSTARTQDRPATVLGAMEIGWSMDLAASKVCMQAFLDRGYREVDTAHYYGEGQSESFLGSMDLGLKDGSKGVKIATKAIP  81
m 182005     1 MSSTARTQDRPATVLGAMEIGWSMDLAASKVCMQAFLDRGYREVDTAHYYGEGQSESFLGSMDLGLKDGSKGVKIATKAIP  81
m 182014     1 MSSAARTQDRPGTVLGAMEMGWRMDLAASKVCMQAFLDRGYREVDTAHYYGEGQSESFLGSMDLGLKDGSKGVKIATKAIP  81
qm 185830    1 MSSAARTQDRPGTVLGAMEMGWRMDLAASKVCMQAFLDRGYREVDTAHYYGEGQSESFLGSMDLGLKDGSKGVKIATKANP  81
m 182043     1 MSSAARTQDRPGTVLGAMEMGWRMDLAASKVCMQAFLDRGYREVDTAHYYGEGQSESFLGSMDLGLKDGSKGVKIATKAIP  81
m 182009     1 MSSTARTQDRPATVLGAMEIGWSMDLAASKVCMQAFLDRGYREVDTAHYYGEGQSESFLGSMDLGLKDGSKGVKIATKAIP  81
qm 185822    1 MSSAARTQDRPGTVLGAMEMGWRMDLAASKVCMQAFLDRGYREVDTAHYYGEGQSESFLGSMDLGLKDGSKGVKIATKAIP  81
qm 185829    1 MSSTARTQDRPATVLGAMEIGWSMDLAASKVCMQAFLDRGYREVDTAHYYGEGQSESFLGSMDLGLKDGSKGVKIATKAIP  81
qm 185839    1 MSSAARTL--PATVLGAMEMGWRMDQASSEASMQAFLDRGHREVDTAFVYGDGQSESFLGSMDLGLKDGSKGVKIATKAIP  79
qm 185811    1 MSSAARTL--PATVLGAMEMGWRMDQASSEASMQAFLDRGHREVDTAFVYGDGQSESFLGSMDLGLKDGSKGVKIATKAIP  79

qm 185823   80 MDGKTLSASSVRFQLETSLKRLQCNRVDLFYLHMPDHNTPVEETLQACNELHKEGKFVELGLSNYASWEVAEICTLCKKNG 160
m 182015    82 MDGKTLSASSVRSQLETSLKRLQCPKVDLFYLHFPDHVTPIEETLEACNELHKEGKFVELGLSNYASWEVAEICTLCTNSG 162
qm 185847   82 LDGKTLSASSVRSQLETSLKRLQCPKVDLFYLHFPDHVTPIEETLEACNELHKEGKFVELGLSNYASWEVAEICTLCKKNG 162
qm 185832   80 MDGKTLSASSVRFQLETSLKRLQCNRVDLFYLHMPDHNTPVEETLQACNELHKEGKFVELGLSNYASWEVAEICTLCTNSG 160
m 182017    82 MDGKTLSASSVRFQLETSLKRLQCNRVDLFYLHMPDHNTPVEETLQACNELHKEGKFVELGLSNYASWEVAEICTLCTNSG 162
m 182026    82 MDGKTLSASSVRFQLETSLKRLQCNRVDLFYLHMPDHNTPVEETLQACNELHKEGKFVELGLSNYASWEVAEICTLCTNSG 162
m 182005    82 MDGKTLSASSVRFQLETSLKRLQCNRVDLFYLHMPDHNTPVEETLQACNELHKEGKFVELGLSNYASWEVAEICTLCTNSG 162
m 182014    82 MDGKTLSASSVRFQLETSLKRLQCNRVDLFYLHMPDHNTPVEETLQACNELHKEGKFVELGLSNYASWEVAEICTLCTNSG 162
qm 185830   82 LDGKTLSASSVRSQLETSLKRLQCPKVDLFYLHFPDHVTPIEETLEACNELHKEGKFVELGLSNYASWEVAEICTLCTNSG 162
m 182043    82 MDGKTLSASSVRFQLETSLKRLQCNRVDLFYLHMPDHNTPVEETLQACNELHKEGKFVELGLSNYASWEVAEICTLCTNSG 162
m 182009    82 MDGKTLSASSVRFQLETSLKRLQCNRVDLFYLHMPDHNTPVEETLQACNELHKEGKFVELGLSNYASWEVAEICTLCTNSG 162
qm 185822   82 MDGKTLSASSVRFQLETSLKRLQCNRVDLFYLHMPDHNTPVEETLQACNELHKEGKFVELGLSNYASWEVAEICTLCTNSG 162
qm 185829   82 MDGKTLSASSVRFQLETSLKRLQCNRVDLFYLHMPDHNTPVEETLQACNELHKEGKFVELGLSNYASWEVAEICTLCTNSG 162
qm 185839   80 MDGKTLSASSVRFQLETSLKRLQCNRVDLFYLHMPDHNTPVEETLQACNELHKEGKFVELGLSNYASWEVAEICTLCTNSG 160
qm 185811   80 MDGKTLSASSVRFQLETSLKRLQCNRVDLFYLHMPDHNTPVEETLQACNELHKEGKFVELGLSNYASWEVAEICTLCTNSG 160

qm 185823  161 WVLPTVYQGMYSAIDRQVEKELFPCLRHYGLRFYVYSPLAGGFLTGKYKYEDKDKKQPITRFFGRRVSQLYHKRYWKVLHF 241
m 182015   163 WVLPTVYQGMYNATTRQVETELFPCLHHYGLRFYVYSPLAGGFLTGKYKYEDKDKKQPITRFFGRRVSQLYHKRYWKVLHF 243
qm 185847  163 WVLPTVYQGMYSAIDRQVEKELFPCLRHYGLRFYVYSPLAGGFLTGKYKYEDKDKKQPITRFFGRRVSQLYHKRYWKVLHF 243
qm 185832  161 WVLPTVYQGMYNATTRQVETELFPCLHHYGLRFYAYNPLAGGLLTGKYRYEDKDKKQPEGRFFGNSWAETYRNRFWKEHHF 241
m 182017   163 WVLPTVYQGMYNATTRQVETELFPCLHHYGLRFYAYNPLAGGLLTGKYRYEDKDKKQPEGRFFGNSWAETYRNRFWKEHHF 243
m 182026   163 WVLPTVYQGMYNATTRQVETELFPCLHHYGLRFYAYNPLAGGLLTGKYRYEDKDKKQPEGRFFGNSWAETYRNRFWKEHHF 243
m 182005   163 WVLPTVYQGMYNAITRQVETELFPCLHRFGLRFYAFNPLAGGLLTGKYTYEDKDKNQPTGRFFGKNLSEIYRNRYWKKHHF 243
m 182014   163 WVLPTVYQGMYNAITRQVETELFPCLHRFGLRFYAFNPLAGGLLTGKYTYEDKDKNQPTGRFFGKNLSEIYRNRYWKKHHF 243
qm 185830  163 WVLPTVYQGMYNAITRQVETELFPCLHRFGLRFYAFNPLAGGLLTGKYNYEDEDDKQPRGRFFQRDFSEHYRNRYWKKHHF 243
m 182043   163 WVLPTVYQGMYNATTRQVETELFPCLHHYGLRFYAYNPLAGGLLTGKYNYEDEDDKQPRGRFFQRDFSEHYRNRYWKKHHF 243
m 182009   163 WVLPTVYQGMYNATTRQVETELFPCLHHYGLRFYAYNPLAGGLLTGKYNYEDEDDKQPRGRFFQRDFSEHYRNRYWKKHHF 243
qm 185822  163 WVLPTVYQGMYNAITRQVETELFPCLHRFGLRFYAFNPLAGGLLTGKYNYEDEDDKQPRGRFFQRDFSEHYRNRYWKKHHF 243
qm 185829  163 WVLPTVYQGMYNAITRQVETELFPCLHRFGLRFYAFNPLAGGLLTGKYNYEDEDDKQPRGRFFQRDFSEHYRNRYWKKHHF 243
qm 185839  161 WVLPTVYQGMYNAITRQVETELFPCLHRFGLRFYAFNPLAGGLLTGKYTYEDKDKNQPTGRFFGKNLSEIYRNRYWKKHHF 241
qm 185811  161 WVLPTVYQGMYNAITRQVETELFPCLHRFGLRFYAFNPLAGGLLTGKYNYEDEDDKQPRGRFFQRDFSEHYRNRYWKKHHF 241

qm 185823  242 QAIDLVRKALAASYGANPPSMTSAALRWMYHHSQIQGARGDAVILGMTSLEQLQQNLAAAEEGPLEPGVVKAFDDAWNLVA 322
m 182015   244 QAIDLVRKALAASYGANPPSMTSAALRWMYHHSKLQGAHGDAVILGMTSLEQLQQNLAAAEEGPLEPGVVKAFDDAWNLVA 324
qm 185847  244 QAIDLVRKALAASYGANPPSMTSAALRWMYHHSQIQGARGDAVILGMTSLEQLQQNLAAAEEGPLEPGVVKAFDDAWNLVA 324
qm 185832  242 QAISLVKKALAASYGASPPSMTSAALRWMYHHSKLQGAHGDAVILGMTSLEQLQQNLAAAEEGPLEPGVVKAFDDAWNLVA 322
m 182017   244 QAISLVKKALAASYGASPPSMTSAALRWMYHHSKLQGAHGDAVILGMTSLEQLQQNLAAAEEGPLEPGVVKAFDDAWNLVA 324
m 182026   244 QAISLVKKALAASYGASPPSMTSAALRWMYHHSKLQGAHGDAVILGMTSLEQLQQNLAAAEEGPLEPGVVKAFDDAWNLVA 324
m 182005   244 QAIDLVKKALVECYGASPPSMTSAALRWMYHHSKLQGAHGDAVILGMTSLEQLQQNLAAAEEGPLEPGVVKAFDDAWNLVA 324
m 182014   244 QAIDLVKKALVECYGASPPSMTSAALRWMYHHSKLQGAHGDAVILGMTSLEQLQQNLAAAEEGPLEPGVVKAFDDAWNLVA 324
qm 185830  244 QAIDLVKKALVECYGASPPSMTSAALRWMYHHSKLQGAHGDAVILGMTSLEQLQQNLAAAEEGPLEPGVVKAFDDAWNLVA 324
m 182043   244 QAIDLVKKALVECYGASPPSMTSAALRWMYHHSKLQGAHGDAVILGMTSLEQLQQNLAAAEEGPLEPGVVKAFDDAWNLVA 324
m 182009   244 QAIDLVKKALVECYGASPPSMTSAALRWMYHHSKLQGAHGDAVILGMTSLEQLQQNLAAAEEGPLEPGVVKAFDDAWNLVA 324
qm 185822  244 QAIDLVKKALVECYGASPPSMTSAALRWMYHHSKLQGAHGDAVILGMTSLEQLQQNLAAAEEGPLEPGVVKAFDDAWNLVA 324
qm 185829  244 QAIDLVKKALVECYGASPPSMTSAALRWMYHHSKLQGAHGDAVILGMTSLEQLQQNLAAAEEGPLEPGVVKAFDDAWNLVA 324
qm 185839  242 KAIDLVKKALAACYETDPPSMTSAALRWMYHHSKLQGAHGDAVILGMTSLEQLQQNLAAAEEGPLEPGVVKAFDDAWNLVA 322
qm 185811  242 QAIDLVKKALVECYGASPPSMTSAALRWMYHHSKLQGAHGDAVILGMTSLEQLQQNLAAAEEGPLEPGVVKAFDDAWNLVA 322

qm 185823  323 HECPSYFR 330
m 182015   325 HECPSYFR 332
qm 185847  325 HECPSYFR 332
qm 185832  323 HECPSYFR 330
m 182017   325 HECPSYFR 332
m 182026   325 HECPSYFR 332
m 182005   325 HECPSYFR 332
m 182014   325 HECPSYFR 332
qm 185830  325 HECPSYFR 332
m 182043   325 HECPSYFR 332
m 182009   325 HECPSYFR 332
qm 185822  325 HECPSYFR 332
qm 185829  325 HECPSYFR 332
qm 185839  323 HECPSYFR 330
qm 185811  323 HECPSYFR 330
```
