# Supplementary material for: A transcriptome resource for the koala (Phascolarctos cinereus): insights into koala retrovirus transcription and sequence diversity
Source: BMC Genomics. 2014 Sep 11;15(1):786. doi: 10.1186/1471-2164-15-786 (PMC4247155; doi:10.1186/1471-2164-15-786)
Supplement: Supplementary file 8 — Additional file 8: Alignment of KoRV gag protein sequences. The first part of the sequence identifier can be used to infer the name of the library from which the sequence was obtained. (PDF 18 KB) [file 12864_2014_6686_MOESM8_ESM.pdf]

```
qm 31459          1 MGQGESTPLSLTLDHWKDVKTRAHNLSVEIRKGKWQTFCSSEWPTFEVGWPPEGTFNPSI 60
11036_composite   1 MGQGESTPLSLTLDHWKDVKTRAHNLSVEIRKGKWQTFCSSEWPTFEVGWPPEGTFNPSI 60
AAF15097          1 MGQGESTPLSLTLDHWKDVKTRAHNLSVEIRKGKWQTFCSSEWPTFEVGWPPEGTFNPSI 60
qm 31546          1 MGQGESTPLSLTLDHWKDVKTRAHNLSVEIRKGKWQTFCSSEWPTFEVGWPPEGTFNPSI 60

qm 31459         61 ISAVKRIVFQETGGHPDQVPYIIVWQDLSNSPPPWVPPLAKIAVASGQDNGRKSAGGRPS 120
11036_composite  61 ISAVKRIVFQETGGHPDQVPYIIVWQDLSNSP----PPLAKIAVASGQDNGRKSAGGRPS 116
AAF15097         61 ISAVKRIVFQETGGHPDQVPYIIVWQDLSNSPPPWVPPLAKIAVASGQDNGRKSAGGRPS 120
qm 31546         61 ISAVKRIVFQETGGHPDQVPYIIVWQDLSNSPPPWVPPLAKIAVASGQDNGRKSAGGRPS 120

qm 31459        121 APSRLPIYPETDSLFLLSEPPPY------PPAPHAVRPPPGLMAEGLGSEGPAAGTRSRR 174
11036_composite 117 APSRLPIYPETDSLFLLSEPPPYPTSPPPPPAPHAARPAPGLMAEGLGSEGPAAGTRSRR 176
AAF15097        121 APSRLPIYPETDSLFLLSEPPPYPTSPPPPPAPHAARPAPGLMAEGLGSEGPAAGTRSRR 180
qm 31546        121 APSRLPIYPETDSLFLLSEPPPYPTSPPPPPAPHAARPAPGRMAEGLGSEGPAAGTRSRR 180

qm 31459        175 PRSPTGDTGPDSTVALPLRAVGPPAEPNGLVPLQYWPFSSADLYNWKSNHPSFSENPTGL 234
11036_composite 177 PRSPTGDTGPDSTVALPLRAVGPPAEPNGLVPLQYWPFSSADLYNWKSNHPSFSENPTGL 236
AAF15097        181 PRSPTGDTGPDSTVALPLRAVGPPAEPNGLVPLQYWPFSSADLYNWKSNHPSFSENPTGL 240
qm 31546        181 PRSPTGDTGPDSTVALPLRAVGPPAEPNGLVPLQYWPFSSADLYNWKSNHPSFSENPTGL 240

qm 31459        235 TGLLESLMFSHQPTWDDCQQLLQVLFTTEERERILLEARKNVLGVNGAPTQLENLINEAF 294
11036_composite 237 TGLLESLMFSHQPTWDDCQQLLQVLFTTEERERILLEARKNVLGVNGAPTQLENLINEAF 296
AAF15097        241 TGLLESLMFSHQPTWDDCQQLLQVLFTTEERERILLEARKNVLGVNGAPTQLENLINEAF 300
qm 31546        241 TGLLESLMFSHQPTWDDCQQLLQVLFTTEERERILLEARKNVLGVNGAPTQLENLINEAF 300

qm 31459        295 PLNRPQWDHNTAEGRERLLVYRRTLVAGLKGAARRPTNLAKVREVLQGPTEPPSVFLERL 354
11036_composite 297 PLNRPQWDHNTAEGRERLLVYRRTLVAGLKGAARRPTNLAKVREVLQGPTEPPSVFLERL 356
AAF15097        301 PLNRPQWDHNTAEGRERLLVYRRTLVAGLKGAARRPTNLAKVREVLQGPTEPPSVFLERL 360
qm 31546        301 PLNRPQWDHNTAEGRERLLVYRRTLVAGLKGAARRPTNLAKVREVLQGPTEPPSVFLERL 360

qm 31459        355 MEAYRRYTPFDPSSEGQKAAVAMSFIGQSAPDIKKKLQRLEGLQDHSLQDLIKEAEKVYH 414
11036_composite 357 MEAYRRYTPFDPSSEGQKAAVAMSFIGQSAPDIKKKLQRLEGLQDHSLQDLIKEAEKVYH 416
AAF15097        361 MEAYRRYTPFDPSSEGQKAAVAMSFIGQSAPDIKKKLQRLEGLQDHSLQDLIKEAEKVYH 420
qm 31546        361 MEAYRRYTPFDPSSEGQKAAVAMSFIGQSAPDIKKKLQRLEGLQDHSLQDLIKEAEKVYH 420

qm 31459        415 KRETEEEKQEREKKETEERERRRDRRQEKNLTKILAAVVSEKGSRGRQAGNLSNRAMRAP 474
11036_composite 417 KRETEEEKQEREKKETEEREREQRDRRQEKNLTKILAAVVSEKGSRGRQAGNLSNRAMRAP 476
AAF15097        421 KRETEEEKQEREKKETEERERRRDRRQEKNLTKILAAVVSEKGFRGRQAGNLSNRAMRAP 480
qm 31546        421 KRETEEEKQEREKKETEERERRRDRRQEKNLTKILAAVVSEKGSRGRQAGNLSNRAMRAP 480

qm 31459        475 REGRPPLDKDQCAYCKERGHWARECPRKKNARETNVLTLGD                    515
11036_composite 477 REGRPPLDKDQCAYCKERGHWARECPRKKNARETNVLTLGD                    517
AAF15097        481 REGRPPLDKDQCAYCKERGHWARECPRKKNARETNVLTLGD                    521
qm 31546        481 REGRPPLDKDQCAYCKERGHWARECPRKKNARETNVLTLGD                    521
```
