# Supplementary material for: A transcriptome resource for the koala (Phascolarctos cinereus): insights into koala retrovirus transcription and sequence diversity
Source: BMC Genomics. 2014 Sep 11;15(1):786. doi: 10.1186/1471-2164-15-786 (PMC4247155; doi:10.1186/1471-2164-15-786)
Supplement: Supplementary file 9 — Additional file 9: Alignment of KoRV pol protein sequences. (PDF 24 KB) [file 12864_2014_6686_MOESM9_ESM.pdf]

```
m 17776      1 MGKMGPKRTVVAGATGSKVYPWTTKRLLKIGQKQVTHSFLVIPECPAPLLGRDLLTKLKA 60
qm 31553     1 MGKMGPKRTVVAGATGSKVYPWTTKRLLKIGQKQVTHSFLVIPECPAPLLGRDLLTKLKA 60
AAF15098     1 MGKMGSKRTVVAGATGSKVYPWTTKRLLKIGQKQVTHSFLVIPECPAPLLGRDLLTKLKA 60

m 17776     61 QIQFSTEGPQVTWEDHPAMCLVLNLEEEYRLHEKPVPPSIDPSWLQLFPMVWAEKAGMGL 120
qm 31553    61 QIQFSTEGPQVTWGDRPAMCLVLNLEEEYRLHEKPVPPSIDPSWLQLFPMVWAEKAGMGL 120
AAF15098    61 QIQFSTEGPQVTWEDRPAMCLVLNLEEEYRLHEKPVPPSIDPSWLQLFPMVWAEKAGMGL 120

m 17776    121 ANQVPPVVVELKSDASPVAVRQYPMSKEAREGIRPHIQRFLDLGILVPCQSPWNTPLLPV 180
qm 31553   121 ANQVPPVVVELKSDASPVAVRQYPMSKEAREGIRPHIQRFLDLGILVPCQSPWNTPLLPV 180
AAF15098   121 ANQVPPVVVELKSDASPVAVRQYPMSKEAREGIRPHIQRFLDLGILVPCQSPWNTPLLPV 180

m 17776    181 KKPGTNDYRPVQDLREVNKRVQDIHPTVPNPYNLLSSLPPSHTWYSVLDLKDAFFCLKLH 240
qm 31553   181 KKPGTNDYRPVQDLREVNKRVQDIHPTVPNPYNLLSSLPPSHTWYSVLDLKDAFFCLKLH 240
AAF15098   181 KKPGTNDYRPVQDLREVNKRVQDIHPTVPNPYNLLSSLPPSHTWYSVLDLKDAFFCLKLH 240

m 17776    241 PNSQPLFAFEWRDPEKGNTGQLTWTRLPQGFKNSPTLFDEALHRDLASFRALNPQVVMLQ 300
qm 31553   241 PNSQPLFAFEWRDPEKGNTGQLTWTRLPQGFKNSPTLFDEALHRDLASFRALNPQVVMLQ 300
AAF15098   241 PNSQPLFAFEWRDPEKGNTGQLTWTRLPQGFKNSPTLFDEALHRDLASFRALNPQVVMLQ 300

m 17776    301 YVDDLLVAAPTYRDCKEGTRRLLQELSKLGYRVSAKKAQLCREEVTYLGYLLKGGKRWLT 360
qm 31553   301 YVDDLLVAAPTYRDCKEGTRRLLQELSKLGYRVSAKKAQLCREEVTYLGYLLKGGKRWLT 360
AAF15098   301 YVDDLLVAAPTYRDCKEGTRRLLQELSKLGYRVSAKKAQLCREEVTYLGYLLKGGKRWLT 360

m 17776    361 PARKATVMKIPTPTTPRQVREFLGTAGFCRLWIPGFASLAAPLYPLTREKVPFTWTEAHQ 420
qm 31553   361 PARKATVMKIPTPTTPRQVREFLGTAGFCRLWIPGFASLAAPLYPLTREKVPFTWTEAHQ 420
AAF15098   361 PARKATVMKIPTPTTPRQVREFLGTAGFCRLWIPGFASLAAPLYPLTREKVPFTWTEAHQ 420

m 17776    421 EAFGRIKEALLSAPALALPDLTKPFALYVDEKEGVARGVLTQTLGPWRRPVAYLSKKLDP 480
qm 31553   421 EAFGRIKEALLSAPALALPDLTKPFALYVDEKEGVARGVLTQTLGPWRRPVAYLSKKLDP 480
AAF15098   421 EAFGRIKEALLSAPALALPDLTKPFALYVDEKEGVARGVLTQTLGPWRRPVAYLSKKLDP 480

m 17776    481 VASGWPTCLKAIAAVALLLKDADKLTLGQNVLVIAPHNLESIVRQPPDRWMTNARMTHYQ 540
qm 31553   481 VASGWPTCLKAIAAVALLLKDADKLTLGQNVLVIAPHNLESIVRQPPDRWMTNARMTHYQ 540
AAF15098   481 VASGWPTCLKAIAAVALLLKDADKLTLGQNVLVIAPHNLESIVRQPPDRWMTNARMTHYQ 540

m 17776    541 SLLLNERVSFAPPAILNPATLLPVESDDTPIHICSEILAEETGTRPDLRDQPLPGVPAWY 600
qm 31553   541 SLLLNERVSFAPPAILNPATLLPVESDDTPIHICSEILAEETGTRPDLRDQPLPGVPAWY 600
AAF15098   541 SLLLNERVSFAPPAILNPATLLPVESDDTPIHICSEILAEETGTRPDLRDQPLPGVPAWY 600

m 17776    601 TDGSSFIMDGRRQAGAAIVDNKRTVWASNLPEGTSAQKAELIALTQALRLAEGKSINIYT 660
qm 31553   601 TDGSSFIMDGRRQAGAAIVDNKRTVWASNLPEGTSAQKAELIALTQALRLAEGKSINIYT 660
AAF15098   601 TDGSSFIMDGRRQAGAAIVDNKRTVWASNLPEGTSAQKAELIALTQALRLAEGKSINIYT 660

m 17776    661 DSRYAFATAHVHGAIYKQRGLLTSAGKDIKNKEEILALLEAIHLPKRVAIIHCPGHQRGT 720
qm 31553   661 DSRYAFATAHVHGAIYKQRGLLTSAGKDIKNKEEILALLEAIHLPKRVAIIHCPGHQRGT 720
AAF15098   661 DSRYAFATAHVHGAIYKQRGLLTSAGKDIKNKEEILALLEAIHLPKRVAIIHCPGHQRGT 720

m 17776    721 DPVATGNRKADEAAKQAAQSTRILTETTKNQEPFEPTRGKIKPRELTPNQGREFIQRLHQ 780
qm 31553   721 DPVATGNRKADEAAKQAAQSTRILTETTKNQEHFEPTRGKIKPRELTPDQGREFLQRLHQ 780
AAF15098   721 DPVATGNRKADEAAKQAAQSTRILTETTKNQEHFEPTRGKIKPRELTPDQGREFIQRLHQ 780

m 17776    781 LTHLGPDKLLQLVGRTSFHIPNLQSVVREITSKCQVCAVTNAVTTYRESGRRQRGDRPGV 840
qm 31553   781 LTHLGPDKLLQLVGRTSFHIPNLQSVVREITSKCQVCAVTNAVTTYRESGRRQRGDRPGV 840
AAF15098   781 LTHLGPDKLLQLVGRTSFHIPNLQSVVREITSKCQVCAVTNAVTTYREPGRRQRGDRPGV 840

m 17776    841 YWEVDFTEVKPGRYGNRYLLVFIDTFSGWVEAFPTKTETALTVCKKILEEILPRFGIPKV 900
qm 31553   841 YWEVDFTEVKPGRYGNRYLLVFIDTFSGWVEAFPTKTETALTVCKKILEEILPRFGIPKV 900
AAF15098   841 YWEVDFTEVKPGRYGNRYLLVFIDTFSGWVEAFPTKTETALTVCKKILEEILPRFGIPKV 900

m 17776    901 LGSDNGPAFVAQVSQGLATQLGINWKLHCAYRPQSSGQVERMNRTIKETLTKLALETGGK 960
qm 31553   901 LGSDNGPAFVAQVSQGLATQLGINWKLHCAYRPQSSGQVERMNRTIKETLTKLALETGGK 960
AAF15098   901 LGSDNGPAFVAQVSQGLATQLGIDWKLHCAYRPQSSGQVERMNRTIKETLTKLALETGGK 960

m 17776    961 DWVTLLPLALLRARNTPGQFGLTPYEILHGGPPPVLASGEVVGSNGDFFPVLFTHLKALE 1020
qm 31553   961 DWVTLLPLALLRARNTPGQFGLTPYEILHGGPPPVLASGEVVGSNGDFFPVLFTHLKALE 1020
AAF15098   961 DWVTLLPLALLRARNTPGQFGLTPYEILHGGPPPVLASGEVVGSNGDFFPVLFTHLKALE 1020

m 17776   1021 VVRTQIWDQIKEAYRPGTVAIPHPFQVGDRVLVRRHRSGSLEPRWKGPYLVLLTTPTAVK 1080
qm 31553  1021 VVRTQIWDQIKEAYRPGTVAIPHPFQVGDRVLVRRHRSGSLEPRWKGPYLVLLTTPTAVK 1080
AAF15098  1021 VVRTQIWDQIKEAYRPGTVAIPHPFQVGDRVLVRRHRSGSLEPRWKGPYLVLLTTPTAVK 1080

m 17776   1081 VDGIAAWVHASHLKPAPPGAPDESWELEKTDHPLKLRVRRRRNESTA              1127
qm 31553  1081 VDGIAAWVHASHLKPAPPGAPDESWELEKTDHPLKLRVRRRRNESTA              1127
AAF15098  1081 VDGIAAWVHASHLKPAPPGAPDESWELEKTDHPLKLRVRRRRNESTA              1127
```
