# Supplementary material for: A transcriptome resource for the koala (Phascolarctos cinereus): insights into koala retrovirus transcription and sequence diversity
Source: BMC Genomics. 2014 Sep 11;15(1):786. doi: 10.1186/1471-2164-15-786 (PMC4247155; doi:10.1186/1471-2164-15-786)
Supplement: Supplementary file 10 — Additional file 10: Alignment of PC and Bi KoRV env protein sequences related to the KoRV-A sequence BAM67147. Two of the sequences (from PC library PC010 and Bi lymph node library) are identical to [GenBank:BAM67147]. (PDF 19 KB) [file 12864_2014_6686_MOESM10_ESM.pdf]

```
PC006_v2_3_comp1_c0_seq7     1 MLLISNPRHLGHPMSPGNWKRLIILLSCVFGGAEMNQQHNNPHQPMTLTWQVLSQTGSVVWEKKAVEPPWTWWPSLEPDV 80
PC010_v2_3_comp0_c0_seq2     1 MLLISNPRHLGHPMSPGNWKRLIILLSCVFGGAEMNQQHNNPHQPMTLTWQVLSQTGSVVWEKKAVEPPWTWWPSLEPDV 80
kidney_v2_3_comp22_c0_seq1   1 MLLISNPRHLGHPMSPGNWKRLIILLSCVFGGAEMNQQHNNPHQPMTLTWQVLSQTGSVVWEKKAVEPPWTWWPSLEPDV 80
PC009b_v2_3_comp2_c0_seq3    1 MLLISNPRHLGHPMSPGNWKRLIILLSCVFGGAEMNQQHNNPHQPMTLTWQVLSQTGSVVWEKKAVEPPWTWWPSLEPDV 80
PC008_v2_3_comp5_c0_seq12    1 MLLISNPRHLGHPMSPGNWKRLIILLSCVFGGAEMNQQHNNPHQPMTLTWQVLSQTGSVVWEKKAVEPPWTWWPSLEPDV 80
PC005_v2_3_comp6_c0_seq3     1 MLLISNPRHLGHPMSPGNWKRLIILLSCVFGGAEMNQQHNNPHQPMTLTWQVLSQTGSVVWEKKAVEPPWTWWPSLEPDV 80

PC006_v2_3_comp1_c0_seq7    81 CALVAGLESWDIPELTASASQQARPPDSNYEHAYNQITWGTLGCSYPRARTRIARSQFYVCPRDGRSLSEARRCGGLESL 160
PC010_v2_3_comp0_c0_seq2    81 CALVAGLESWDIPELTASASQQARPPDSNYEHAYNQITWGTLGCSYPRARTRIARSQFYVCPRDGRSLSEARRCGGLESL 160
kidney_v2_3_comp22_c0_seq1  81 CALVAGLESWDIPELTASASQQARPPDSNYEHAYNQITWGTLGCSYPRARTRIARSQFYVCPRDGRSLSEARRCGGLESL 160
PC009b_v2_3_comp2_c0_seq3   81 CALVAGLESWDIPELTASASQQARPPDSNYEHAYNQITWGTLGCSYPRARTRIARSQFYVCPRDGRPLSEARRCGGLESL 160
PC008_v2_3_comp5_c0_seq12   81 CALVAGLESWDIPELTASASQQARPPDSNYEHAYNQITWGTLGCSYPRARTRIARSQFYVCPRDGRSLSEARRCGGLESL 160
PC005_v2_3_comp6_c0_seq3    81 CALVAGLESWDIPELTASASQQARPPDSNYEHAYNQITWGTLGCSYPRARTRIARSQFYVCPRDGRSLSEARRCGGLESL 160

PC006_v2_3_comp1_c0_seq7   161 YCKEWGCETAGTAYWQPRSSWDLITVVQGHPTGTCERTGWCNPLKIEFTEPGKRFRNWLQGRTWGLRFYVTGHPGVQLTI 240
PC010_v2_3_comp0_c0_seq2   161 YCKEWGCETAGTAYWQPRSSWDLITVGQGHPTGTCERTGWCNPLKIEFTEPGKRFRNWLQGRTWGLRFYVTGHPGVQLTI 240
kidney_v2_3_comp22_c0_seq1 161 YCKEWGCETAGTAYWQPRSSWDLITVGQGHPTGTCERTGWCNPLKIEFTEPGKRFRNWLQGRTWGLRFYVTGHPGVQLTI 240
PC009b_v2_3_comp2_c0_seq3  161 YCKEWGCETAGTAYWQPRSSWDLITVGQGHPTGTCERTGWCNPLKIEFTEPGKRFRNWLQGRTWGLRFYVTGHPGVQLTI 240
PC008_v2_3_comp5_c0_seq12  161 YCKEWGCETAGNAYWQPRSSWDLITVGQGHPTGTCERTGWCNPLKIEFTEPGKQFRNWLQGRTWGLRFYVTGHPGVQLTI 240
PC005_v2_3_comp6_c0_seq3   161 YCKEWGCETAGNAYWQPRSSWDLITVGQGHPTGTCERTGWCNPLKIEFTEPGKQFRNWLQGRTWGLRFYVTGHPGVQLTI 240

PC006_v2_3_comp1_c0_seq7   241 RLVITSPPPVVVGPDPVLAEQGPPRKIPFLPRVPVPTLSPPASPIPTVQASPPAPSTPSPTTGDRLFGLVQGAFLALNAT 320
PC010_v2_3_comp0_c0_seq2   241 RLVITSPPPVVVGPDPVLAEQGPPRKIPFLPRVPVPTLSPPASPIPTVQASPPAPSTPSPTTGDRLFGLVQGAFLALNAT 320
kidney_v2_3_comp22_c0_seq1 241 RLVITSPPPVVVGPDPVLAEQGPPRKIPFLPRVPVPTLSPPASPIPTVQASPPAPSTPSPTTGDRLFGLVQGAFLALNAT 320
PC009b_v2_3_comp2_c0_seq3  241 RLVITSPPPVVVGPDPVLAEQGPPRKIPFLPRVPVPTLSPPASPIPTVQASPPAPSTPSPTTGDRLFGLVQGAFLALNAT 320
PC008_v2_3_comp5_c0_seq12  241 RLVITSPPPVVVGPDPVLAEQGPPRKIPFLPRVPVPTLSPPASPIPTVQASPPAPSTPSPTTGDRLFGLVQGAFLALNAT 320
PC005_v2_3_comp6_c0_seq3   241 RLVITSPPPVVVGPDPVLAEQGPPRKIPFLPRVPVPTLSPPASPIPTVQASPPAPSTPSPTTGDRLFGLVQGAFLALNAT 320

PC006_v2_3_comp1_c0_seq7   321 NPEATESCWLCLALGPPYYEGIATPGQVTYASTDSQCRWGGKGKLTLTEVSGLGLCIGKVPPTHQHLCNLTIPLNASHTH 400
PC010_v2_3_comp0_c0_seq2   321 NPEATESCWLCLALGPPYYEGIATPGQVTYASTDSQCRWGGKGKLTLTEVSGLGLCIGKVPPTHQHLCNLTIPLNASHTH 400
kidney_v2_3_comp22_c0_seq1 321 NPEATESCWLCLALGPPYYEGIATPGQVTYASTDSQCRWGGKGKLTLTEVSGLGLCIGKVPPTHQHLCNLTIPLNASHTH 400
PC009b_v2_3_comp2_c0_seq3  321 NPEATESCWLCLALGPPYYEGIATPGQVTYASTDSQCRWGGKGKLTLTEVSGLGLCIGKVPPTHQHLCNLTIPLNASHTH 400
PC008_v2_3_comp5_c0_seq12  321 NPEATESCWLCLALGPPYYEGIATPGQVTYASTDSQCRWGGKGKLTLTEVSGLGLCIGKVPPTHQHLCSLTIPLNASHTH 400
PC005_v2_3_comp6_c0_seq3   321 NPEATESCWLCLALGPPYYEGIATPGQVTYASTDSQCRWGGKGKLTLTEVSGLGLCIGKVPPTHQHLCNLTIPLNASHTH 400

PC006_v2_3_comp1_c0_seq7   401 KYLLPSNHSWWACNSGLTPCLSTSVFNQSNDFCIQIQLVPRIYYHPDGTLLQAYESPHPRNKREPVSLTLAVLLGLGVAA 480
PC010_v2_3_comp0_c0_seq2   401 KYLLPSNHSWWACNSGLTPCLSTSVFNQSNDFCIQIQLVPRIYYHPDGTLLQAYESPHPRNKREPVSLTLAVLLGLGVAA 480
kidney_v2_3_comp22_c0_seq1 401 KYLLPSNHSWWACNSGLTPCLSTSVFNQSNDFCIQIQLVPRIYYHPDGTLLQAYESPHPRNKREPVSLTLAVLLGLGVAA 480
PC009b_v2_3_comp2_c0_seq3  401 KYLLPSNHSWWACNSGLTPCLSTSVFNQSNDFCIQIQLVPRIYYHPDGTLLQAYESPHPRNKREPVSLTLAVLLGLGVAA 480
PC008_v2_3_comp5_c0_seq12  401 KYLLPSNHSWWACNSGLTPCLSTSVFNQSNDFCIQIQLVPRIYYHPDGTLLQAYESPHPRNKREPVSLTLAVLLGLGVAA 480
PC005_v2_3_comp6_c0_seq3   401 KYLLPSNHSWWACNSGLTPCLSTSVFNQSNDFCIQIQLVPRIYYHPDGTLLQAYESPHPRNKREPVSLTLAVLLGLGVAA 480

PC006_v2_3_comp1_c0_seq7   481 GIGTGSTALIKGPIDLQQGLTSLQIAMDTDLRALQDSISKLEDSLTSLSEVVLQNRRGLDLLFLKEGGLCAALKEECCFY 560
PC010_v2_3_comp0_c0_seq2   481 GIGTGSTALIKGPIDLQQGLTSLQIAMDTDLRALQDSISKLEDSLTSLSEVVLQNRRGLDLLFLKEGGLCAALKEECCFY 560
kidney_v2_3_comp22_c0_seq1 481 GIGTGSTALIKGPIDLQQGLTSLQIAMDTDLRALQDSISKLEDSLTSLSEVVLQNRRGLDLLFLKEGGLCAALKEECCFY 560
PC009b_v2_3_comp2_c0_seq3  481 GIGTGSTALIKGPIDLQQGLTSLQIAMDTDLRALQDSISKLEDSLTSLSEVVLQNRRGLDLLFLKEGGLCAALKEECCFY 560
PC008_v2_3_comp5_c0_seq12  481 GIGTGSTALIKGPIDLQQGLTSLQIAMDTDLRALQDSISKLEDSLTSLSEVVLQNRRGLDLLFLKEGGLCAALKEECCFY 560
PC005_v2_3_comp6_c0_seq3   481 GIGTGSTALIKGPIDLQQGLTSLQIAMDTDLRALQDSISKLEDSLTSLSEVVLQNRRGLDLLFLKEGGLCAALKEECCFY 560

PC006_v2_3_comp1_c0_seq7   561 VDHSGAVRDSMRRLKERLDKRQLEHQKNLSWYEGWFNRSPWLTTLLSALAGPLLLLLLLLTLGPCVINKLVQFINDRVSA 640
PC010_v2_3_comp0_c0_seq2   561 VDHSGAVRDSMRRLKERLDKRQLEHQKNLSWYEGWFNRSPWLTTLLSALAGPLLLLLLLLTLGPCVINKLVQFINDRVSA 640
kidney_v2_3_comp22_c0_seq1 561 VDHSGAVRDSMRRLKERLDKRQLEHQKNLSWYEGWFNRSPWLTTLLSALAGPLLLLLLLLTLGPCVINKLVQFINDRVSA 640
PC009b_v2_3_comp2_c0_seq3  561 VDHSGAVRDSMRRLKERLDKRQLEHQKNLSWYEGWFNRSPWLTTLLSALAGPLLLLLLLLTLGPCVINKLVQFINDRVSA 640
PC008_v2_3_comp5_c0_seq12  561 VDHSGAVRDSMRRLKERLDKRQLEHQKNLSWYEGWFNRSPWLTTLLSALAGPLLLLLLLLTLGPCVINKLVQFINDRVSA 640
PC005_v2_3_comp6_c0_seq3   561 VDHSGAVRDSMRRLKERLDKRQLEHQKNLSWYEGWFNRSPWLTTLLSALAGPLLLLLLLLTLGPCVINKLVQFINDRVSA 640

PC006_v2_3_comp1_c0_seq7   641 VRILVLRHKYQTLDNEDNL 659
PC010_v2_3_comp0_c0_seq2   641 VRILVLRHKYQTLDNEDNL 659
kidney_v2_3_comp22_c0_seq1 641 VRILVLRHKYQTLDNEDNL 659
PC009b_v2_3_comp2_c0_seq3  641 VRILVLRHKYQTLDNEDNL 659
PC008_v2_3_comp5_c0_seq12  641 VRILVLRHKYQTLDNEDNL 659
PC005_v2_3_comp6_c0_seq3   641 VRILVLRHKYQTLDNEDNL 659
```
