# Supplementary material for: A transcriptome resource for the koala (Phascolarctos cinereus): insights into koala retrovirus transcription and sequence diversity
Source: BMC Genomics. 2014 Sep 11;15(1):786. doi: 10.1186/1471-2164-15-786 (PMC4247155; doi:10.1186/1471-2164-15-786)
Supplement: Supplementary file 11 — Additional file 11: Alignment of PC and Bi KoRV env protein sequences with the KoRV-A sequence [GenBank:BAN63360]. (PDF 20 KB) [file 12864_2014_6686_MOESM11_ESM.pdf]

```
BAN63360                          1 MLLISNPRHLGHPMSPGNWKRLIIILLSCVFGGAEMNQQHNNPHQPMTLTWQVLSQTGSVVWEKKAVEPPWTWWPSLEPDV 80
lymphnode_v2_3_comp821_c0_seq2    1 MLLISNPRHPGHPMSPGNWKRLIIILLSCVFGGAEMNRQHNNPHQPMTLTWQVLSQTGSVVWEKKAVEPPWTWWPSLEPDV 80
PC001_v2_3_comp14_c0_seq15        1 MLLISNPRHLGHPMSPGNWKRLIIILLSCVFGGAEMNQQHNNPHQPMTLTWQILSQTGSVVWEKKAVEPPWTWWPSLEPDV 80

BAN63360                         81 CALVAGLESWDIPELTASASQQARPPLYDRPWGTPGAGFTAAGWGGTLGCSYPRARIRMAMSQFYVCPRDGRSLSEARRC 160
lymphnode_v2_3_comp821_c0_seq2   81 CALVAGLESWDIPELTASASQQARPPLYDRPWGTPGAGFAAAGWGGTLGCSYPRARTRIAMSPFYVCPRDGRSLSEARRC 160
PC001_v2_3_comp14_c0_seq15       81 CALVAGLESWDIPELTASASQQARPPLYDRPWGTPGAGFAAAGWGGTLGCSYPRARTRISMSQFYVCPRDGRSLSEARRC 160

BAN63360                        161 GGLESLFCKRWGCETAGTAYWQPRSSWDLITVGRGYPTGRTCEHTGWCNPLKIEFTEPGKQSRNWLQGRTWGLRFYVTGH 240
lymphnode_v2_3_comp821_c0_seq2  161 GGLESLYCKEWGCETAGTAYWQPRSSWDLITVGRGHPTG-TCEHTGWCNPLKIEFTEPGKRFRNWLQGRTWGLRFYVTGH 239
PC001_v2_3_comp14_c0_seq15      161 GGLESLYCKKWGCETAGNAYWQPRSSWDLITVGRGHPTG-TCESTGWCNPLKIEFTEPGKQFRNWLQGRTWGLRFYVTGH 239

BAN63360                        241 PGVQLTIRLVITSPPPVLLGPDSVLAEQGPPRKIPFLPRVPVPTLSPPASPIPTVQASPPAPSTPSPTTGDRLFGLVQGA 320
lymphnode_v2_3_comp821_c0_seq2  240 PGVQLTIRLVITSPPPVVVGPDPVLAEQGPPREIPFLPRVPVPTLSPPASPIPTVQASPPAPSTPSPTTGDRLFGLVQGA 319
PC001_v2_3_comp14_c0_seq15      240 PGVQLTIRLVITSPPPVGVGPDPVLAEQGPPRKIPFLPRVPVPTLSPPASPIPTVQASLPTPSTPSPTTGDRLFGLVQGA 319

BAN63360                        321 FLALNATNPEATESCWLCLALGPPYYEGIATPGQVTYASTDSQCRWGGKGKLTLTEVSGLGLCIGKVPPTHQHLCSLTIP 400
lymphnode_v2_3_comp821_c0_seq2  320 FLALNATNPEATESCWLCLALAPPYYEGIATPGQVTYASTDSQCRWGGKGKLTLTEVSGLGLCIGKVPPTHQHLCNLTIP 399
PC001_v2_3_comp14_c0_seq15      320 FLALNATNPEATESCWLCLALGPPYYEGIATPGQVTYASTDSQCRWGGKGKLTLTEVSGLGLCIGKVPPTHQHLCSLTIP 399

BAN63360                        401 LNVSHTHKYLLPSNHGWWACSSGLTPCLSTSVFNQSNDFCIQIQLVPRIYYHPDGTLLQAYESPHPRNKREPVSLTLAVL 480
lymphnode_v2_3_comp821_c0_seq2  400 LNASHTHKYLLPSNHSWWACNSGLTPCLSTSVFNQSNDFCIQIQLVPRIYYHPDGTLLQAYESPHPRHKREPVSLTLAVL 479
PC001_v2_3_comp14_c0_seq15      400 LNASHTHKYLLPSNHSWWACNSGLTPCLSTSVFNQSNDFCIQIQLVPRIYYHPDGTLLQAYESPHPRNKREPVSLTLAVL 479

BAN63360                        481 LGLGVAAGIGTGSTALIKGPIDLQQGLTSLQIAMDTDLRALQDSVSKLEDSLTSLSEVVLQNRRGLDLLFLKEGGLCAAL 560
lymphnode_v2_3_comp821_c0_seq2  480 LGLGVAAGIGTGSTALIKGPIDLQQGLTSLQIAMDTDLRALQDSISKLEDSLTSLSEVVLQNRRGLDLLFLKEGGLCAAL 559
PC001_v2_3_comp14_c0_seq15      480 LGLGVAAGIGTGSTALIKGPIDLQQGLTSLQIAMDTDLRALQDSVSKLEDSLTSLSEVVLQNRRGLDLLFLKEGGLCAAL 559

BAN63360                        561 KEECCFYVDHSGAVRDSMRRLKERLDKRQLEHQKNLSWYEGWFNRSPWLTTLLSALAGPLLLLLLLLTLGPCVINKLVQF 640
lymphnode_v2_3_comp821_c0_seq2  560 KEECCFYVDHSGAVRDSMRRLKERLDKRQLEHQKNLSWYEGWFNRSPWLTTLLSALAGPLLLLLLLLTLGPCVINKLVQF 639
PC001_v2_3_comp14_c0_seq15      560 KEECCFYVDHSGAVRDSMRRLKERLDKRQLEHQKNLSWYEGWFNRSPWLTTLLSALAGPLLLLLLLLTLGPCVINKLVQF 639

BAN63360                        641 INDRVSAVRILVLRHRYQTLDNEDNL 666
lymphnode_v2_3_comp821_c0_seq2  640 INDRVSAVRILVLRHKYQTLDNEDNL 665
PC001_v2_3_comp14_c0_seq15      640 INDRVSAVRILVLRHKYQTLDNEDNL 665
```
