# Supplementary material for: A transcriptome resource for the koala (Phascolarctos cinereus): insights into koala retrovirus transcription and sequence diversity
Source: BMC Genomics. 2014 Sep 11;15(1):786. doi: 10.1186/1471-2164-15-786 (PMC4247155; doi:10.1186/1471-2164-15-786)
Supplement: Supplementary file 12 — Additional file 12: Alignment of a PC KoRV env protein sequence with the KoRV-B sequence [GenBank:AGO86848]. (PDF 18 KB) [file 12864_2014_6686_MOESM12_ESM.pdf]

```
PC008_v2_3_comp5_c0_seq13    1 MLLISNPRHLGHPMSPGNWKRLIIILLSCVFGGAEMNQQHNNPHQPMTLTWQVLSQTGSVVWEKKAVEPPWTWWPSLEPDV 80
AGO86848                     1 MLLISNPRHLGHPMSPGNWKRLIIILLSCVFGGAEMNQQHNNPHQPMTLTWQVLSQTGSVVWEKKAVEPPWTWWPSLEPDV 80

PC008_v2_3_comp5_c0_seq13   81 CALVAGLESWDIPELTASDSQRPKPPGSSCGVSYSQVGRGLVRQTSYGTLGCRCPKDRARFSGFGFYVCPRDGRTLSEAR 160
AGO86848                    81 CALAAGLETWGIPHLTAPESQQSAPP-DGCGVLYSQVGRGLVRQSSYGTLGCRCPRDRNRLAQSQFYVCPRDGRSLSEAW 159

PC008_v2_3_comp5_c0_seq13  161 RCGGLESLYCKEWGCETAGNAYWQPRSSWDLITVGQGHPTGTCERTGWCNPLKIEFTEPGKQFRNWLQGRTWGLRFYVTG 240
AGO86848                   160 RCGGFESLYCKEWGCETTGTAYWQPRSSWDLITVGLGHPTGTCEHTGWCNPLKIEFTEPGKRFRNWLQGRTWGLRFYVTG 239

PC008_v2_3_comp5_c0_seq13  241 HPGVQLTIRLVITSPPPVVVGPDPVLAEQGPPRKIPFLPRVPVPTLSPPASPIPTVQASPPAPSTPSPTTGDRLFGLVQG 320
AGO86848                   240 HPGVQLTIRLVITSPPPVVVGPDPVLAEQGPPRKIPFLPRVPVPTLSPPASPIPTVQASPPAPSTPSPTTGDRLFGLVQG 319

PC008_v2_3_comp5_c0_seq13  321 AFLALNATNPEATESCWLCLALGPPYYEGIATPGQVTYASTDSQCRWGGKGKLTLTEVSGLGLCIGKVPPTHQHLCSLTI 400
AGO86848                   320 AFLALNATNPEATESCWLCLALGPPYYEGIATPGQVTYASTDSQCRWGGKGKLTLTEVSGLGLCIGKVPPTHQHLCNLTI 399

PC008_v2_3_comp5_c0_seq13  401 PLNASHTHKYLLPSNHSWWACNSGLTPCLSTSVFNQSNDFCIQIQLVPRIYYHPDGTLLQAYESPHPRNKREPVSLTLAV 480
AGO86848                   400 PLNASHTHKYLLPSNHSWWACNSGLTPCLSTSVFNQSNDFCIQIQLVPRIYYHPDGTLLQAYESPHPRNKREPVSLTLAV 479

PC008_v2_3_comp5_c0_seq13  481 LLGLGVAAGIGTGSTALIKGPIDLQQGLTSLQIAMDTDLRALQDSISKLEDSLTSLSEVVLQNRRGLDLLFLKEGGLCAA 560
AGO86848                   480 LLGLGVAAGIGTGSTALIKGPIDLQQGLTSLQIAMDTDLRALQDSVSKLENSLTSLSEVVLQNRRGLDLLFLKEGGLCAA 559

PC008_v2_3_comp5_c0_seq13  561 LKEECCFYVDHSGAVRDSMRRLKERLDKRQLEHQKNLSWYEGWFNRSPWLTTLLSALAGPLLLLLLLLTLGPCVINKLVQ 640
AGO86848                   560 LKEECCFYVDHSGAVRDSMRRLKERLDKRQLEHQKNLSWYEGWFNRSPWLTTLLSALAGPLLLLLLLLTLGPCVINKLVQ 639

PC008_v2_3_comp5_c0_seq13  641 FINDRVSAVRILVLRHKYQTLDNEDNL 667
AGO86848                   640 FINDRVSAVRILVLRHKYQTLDNEDNL 666
```
